# CH_4_ and N_2_O emissions from smallholder agricultural systems on tropical peatlands in Southeast Asia

**DOI:** 10.1111/gcb.16747

**Published:** 2023-05-15

**Authors:** Antonio Jonay Jovani‐Sancho, Patrick O'Reilly, Gusti Anshari, Xin Yi Chong, Neil Crout, Christopher D. Evans, Stephanie Evers, Jing Ye Gan, Christopher N. Gibbins, Evi Gusmayanti, Jamaludin Jamaludin, Adi Jaya, Susan Page, Yosep Yosep, Caroline Upton, Paul Wilson, Sofie Sjögersten

**Affiliations:** ^1^ School of Biosciences University of Nottingham Loughborough UK; ^2^ UK Centre for Ecology & Hydrology Bangor UK; ^3^ School of Geography, Geology & the Environment University of Leicester Leicester UK; ^4^ School of Biological and Environmental Sciences Liverpool John Mores University Liverpool UK; ^5^ Magister of Environmental Science Universitas Tanjungpura Pontianak Indonesia; ^6^ Soil Science Department Universitas Tanjungpura Pontianak Indonesia; ^7^ School of Environmental and Geographical Sciences University of Nottingham Malaysia Semenyih Malaysia; ^8^ Agrotechnology Department Universitas Tanjungpura Pontianak Indonesia; ^9^ Universitas Nahdlatul Ulama, Kabupaten Kubu Raya Kalimantan Barat Indonesia; ^10^ Faculty of Agriculture University of Palangka Raya Palangka Raya Indonesia

**Keywords:** agriculture, crop production, emission factors, greenhouse gas fluxes, methane, nitrous oxide, peat swamp forest, tropical peatlands

## Abstract

There are limited data for greenhouse gas (GHG) emissions from smallholder agricultural systems in tropical peatlands, with data for non‐CO_2_ emissions from human‐influenced tropical peatlands particularly scarce. The aim of this study was to quantify soil CH_4_ and N_2_O fluxes from smallholder agricultural systems on tropical peatlands in Southeast Asia and assess their environmental controls. The study was carried out in four regions in Malaysia and Indonesia. CH_4_ and N_2_O fluxes and environmental parameters were measured in cropland, oil palm plantation, tree plantation and forest. Annual CH_4_ emissions (in kg CH_4_ ha^−1^ year^−1^) were: 70.7 ± 29.5, 2.1 ± 1.2, 2.1 ± 0.6 and 6.2 ± 1.9 at the forest, tree plantation, oil palm and cropland land‐use classes, respectively. Annual N_2_O emissions (in kg N_2_O ha^−1^ year^−1^) were: 6.5 ± 2.8, 3.2 ± 1.2, 21.9 ± 11.4 and 33.6 ± 7.3 in the same order as above, respectively. Annual CH_4_ emissions were strongly determined by water table depth (WTD) and increased exponentially when annual WTD was above −25 cm. In contrast, annual N_2_O emissions were strongly correlated with mean total dissolved nitrogen (TDN) in soil water, following a sigmoidal relationship, up to an apparent threshold of 10 mg N L^−1^ beyond which TDN seemingly ceased to be limiting for N_2_O production. The new emissions data for CH_4_ and N_2_O presented here should help to develop more robust country level ‘emission factors’ for the quantification of national GHG inventory reporting. The impact of TDN on N_2_O emissions suggests that soil nutrient status strongly impacts emissions, and therefore, policies which reduce N‐fertilisation inputs might contribute to emissions mitigation from agricultural peat landscapes. However, the most important policy intervention for reducing emissions is one that reduces the conversion of peat swamp forest to agriculture on peatlands in the first place.

## Graphical abstract

The aim of this study was to quantify soil CH_4_ and N_2_O fluxes from smallholder agricultural systems on tropical peatlands in Southeast Asia and assess their environmental controls. Annual CH_4_ emissions were strongly determined by water table depth (WTD) and increased exponentially when annual WTD was above −25 cm. In contrast, annual N_2_O emissions were strongly correlated with mean total dissolved nitrogen (TDN) in soil water. The impact of TDN on N_2_O emissions suggests that soil nutrient status strongly impacts emissions, and therefore, policies which reduce N‐fertilisation inputs might contribute to emissions mitigation from agricultural peat landscapes.

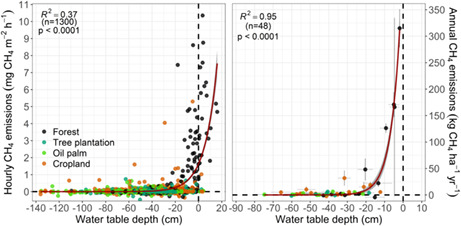

## Abstrak

Data gas rumah kaca, terutama gas metana dan nitrogen, dari kegiatan pertanian skala kecil pada lahan gambut tropis sangat jarang diteliti. Tujuan penelitian ini untuk mengukur emisi gas CH_4_ dan N_2_O dari pertanian skala kecil pada lahan gambut di Asia Tenggara dan mempelajari faktor‐faktor lingkungan yang berhubungan dengan emisi kedua gas tersebut. Riset dilaksanakan pada empat tipe penggunaan lahan di Malaysia dan Indonesia. Fluks CH_4_ dan N_2_O dan parameter lingkungan diukur dari pertanian hortikultura, kebun kelapa sawit, kebun campuran (karet dan jelutung), dan hutan sekunder. Emisi tahunan gas metana (kg CH_4_ per ha per tahun) sebesar 70.7 ± 29.5; 2.1 ± 1.2; 2.1 ± 0.6 dan 6.2 ± 1.9 dari lahan hutan, kebun campuran, kebun kelapa sawit, dan pertanian hortikultura. Dan emisi gas nitrogen (kg N_2_O per ha per tahun) dari empat penggunaan lahan di atas adalah 6.5 ± 2.8; 3.2 ± 1.2; 21.9 ± 11.4 dan 33.6 ± 7.3. Ketinggian muka air tanah sangat mempengaruhi emisi gas metana, dan besarnya emisi tahunan gas metana meningkat secara eksponensial pada tinggi muka air tanah sebesar lebih dari −25 cm. Total nitrogen terlarut (TNT) dalam air tanah gambut sangat berkorelasi dengan emisi gas N_2_O, membentuk hubungan sigmoid. Konsentrasi 10 mg N per liter menjadi faktor pembatas, ketika produksi N_2_O berkurang. Pemerintah dapat menggunakan data baru emisi metana dan nitrogen untuk memperbaiki nilai faktor emisi untuk melaporkan emisi gas‐gas rumah kaca skala nasional. Status hara, terutama dampak pemupukan, berperanan terhadap peningkatan emisi. Karena itu, pemakaian pupuk yang efisien dan pengurangan pemupukan perlu menjadi pertimbangan dan menjadi satu langkah penting untuk mengurangi emisi gas‐gas rumah kaca. Yang terpenting adalah pencegahan perubahan hutan rawa gambut di Asia Tenggara menjadi penggunaan lain, seperti pengembangan pertanian, merupakan upaya prioritas untuk mengurangi emisi gas‐gas rumah kaca dari lahan gambut.

## 1 INTRODUCTION

Tropical peatlands in their natural state are highly effective long‐term carbon (C) stores (Cooper et al., 2020; Deshmukh et al., 2020; Mishra et al., 2021; Prananto et al., 2020). However, when they are converted to other land uses such as agriculture or plantations, they become significant sources of greenhouse gases (GHGs), especially CO_2_ and N_2_O (Page et al., 2022 and references therein). While CO_2_ is the main GHG emitted from drained tropical peatlands (Cooper et al., 2020; Deshmukh et al., 2021) and has been studied more intensively, peat surface CH_4_ and N_2_O emissions have received comparatively little attention. Soil GHG emissions are controlled by a range of factors, with the main ones being groundwater level, temperature and organic matter quality (Couwenberg et al., 2010; Evans et al., 2021; Leifeld et al., 2012; Mishra et al., 2021). As these factors are strongly modified by conversion of peat swamp forest (PSF) to plantations (such as oil palm or Acacia) or other forms of agriculture, such changes have important implications for net GHG emissions. For example, emissions from tropical peatlands converted to agriculture contribute substantially to national emissions of GHGs in Indonesia and Malaysia (Cooper et al., 2020; Miettinen et al., 2017). These increases in GHG emissions are caused by loss of peat‐forming swamp forest cover, drainage for cultivation, and use of fertilisers, which in turn change the soil C compound composition and microbial soil communities (Oktarita et al., 2017; Prananto et al., 2020).

To date, research on the impacts of peatland drainage and conversion to plantations and agriculture have been carried out both in large‐scale plantations and smallholder systems (Deshmukh et al., 2020; Matysek et al., 2018; McCalmont et al., 2021; Miettinen et al., 2016). However, more research on the complex small‐scale agricultural production system on converted peatlands is needed as it is an important land use in the region (Hadi et al., 2005; Inubushi et al., 2003; Swails et al., 2021). Small‐scale agricultural production is responsible for around 44% of the drainage and conversion of peatlands, occupying a similar area of peatland to that incorporated into large‐scale production (Miettinen et al., 2016; Wijedasa et al., 2018). In Indonesia, oil palm plantations cover around 14.6 Mha with approximately 41% of the total area identified as small‐scale plantations and 3–4 Mha established on peatlands (BPS, 2020). Due to global market demands and shortage of available mineral soil for farming, it is expected that the expansion of smallholder agriculture on peatlands will increase (Euler et al., 2016; Jelsma et al., 2017), and it may become a regionally important source of emissions (Jauhiainen et al., 2012). However, small‐scale production practices such as soil amendments and fertiliser applications are very diverse (Table S1), and they may differ from larger scale plantations in many critical respects (e.g. with regards to crop selection, soil management, nutrient application, drainage control and use of fire). A lack of research into smallholder production systems means that C losses and GHG emissions from such systems are poorly understood and emission factors (EFs, i.e. average emissions of each GHG associated with that land use; IPCC, 2006, 2014) for these systems have not been quantified. Our lack of understanding of smallholder agricultural systems represents an important knowledge gap, and emissions data obtained from industrial plantations are unlikely to provide a reliable proxy given differences in management. In particular, there is a need for specific EFs for CH_4_ and N_2_O that are both important GHGs in the context of conversion of tropical peat swamp forest to agriculture (Jauhiainen et al., 2012).

Ground water levels are a first order determinant of the functioning of peatlands; controlling GHG production and release (Couwenberg et al., 2010; Evans et al., 2021; Evers et al., 2017). Under anaerobic conditions, methanogenic bacteria produce CH_4_ (Jauhiainen et al., 2016). In contrast, CH_4_ emissions from the peat surface are near‐zero when groundwater levels are below −30 cm (Couwenberg et al., 2010; Evans et al., 2021). The effect of water table depth (WTD) on N_2_O emissions on tropical peatlands has, thus far, lacked extensive research attention. Combining data from a wide range of sites, Prananto et al. (2020) found higher N_2_O emissions from sites with deeper WTD. However, they did not disentangle the effects of drainage from fertiliser application practices. Indeed, drained agricultural sites are often managed more intensively and receive higher inputs of fertilisers, compared to undrained peatlands, confounding these two factors. Results from other locations and ecosystems have also shown contradictory results between soil moisture content and N_2_O emissions (Couwenberg et al., 2011; Pärn et al., 2018). This suggests that the groundwater level response is governed by site/system‐specific factors, likely to be mainly determined by levels of N inputs, and that relationships between WTD and N_2_O emissions are not generic. In addition, degradation without drainage and fertiliser inputs also increases soil N_2_O emissions (Swails et al., 2021). The interaction of higher soil temperature, optimum soil moisture content and high mineral N concentrations, resulting from fertilisation and peat mineralisation, could convert tropical peatlands into hotspots of N_2_O emissions (Pärn et al., 2018).

The aim of this study was to quantify soil CH_4_ and N_2_O fluxes from different agricultural landscapes on tropical peatlands in Southeast Asia, comparing three typical forms of smallholder farm production systems (short rotation agricultural crops, oil palm plantation and tree plantation) with adjacent forest. Specific research questions were: (1) What are the net annual CH_4_ and N_2_O fluxes from different smallholder land uses compared to adjacent forest areas? (2) How do soil temperature, groundwater level and total dissolved nitrogen (TDN) influence CH_4_ and N_2_O fluxes? (3) Can CH_4_ and N_2_O fluxes in tropical peatlands, for a broad range of vegetation groups and environmental conditions, be reliably predicted from the land‐use class and/or environmental factors? To address these questions at a regionally relevant scale, we undertook a field‐based study at four distinct regions in Malaysia and Indonesia. In each region, we conducted measurements of CH_4_ and N_2_O fluxes along with environmental conditions from three to four different land‐use classes in situ over a 12‐month period.

## 2 MATERIALS AND METHODS

### 2.1 Study area and site selection

**FIGURE 1:**
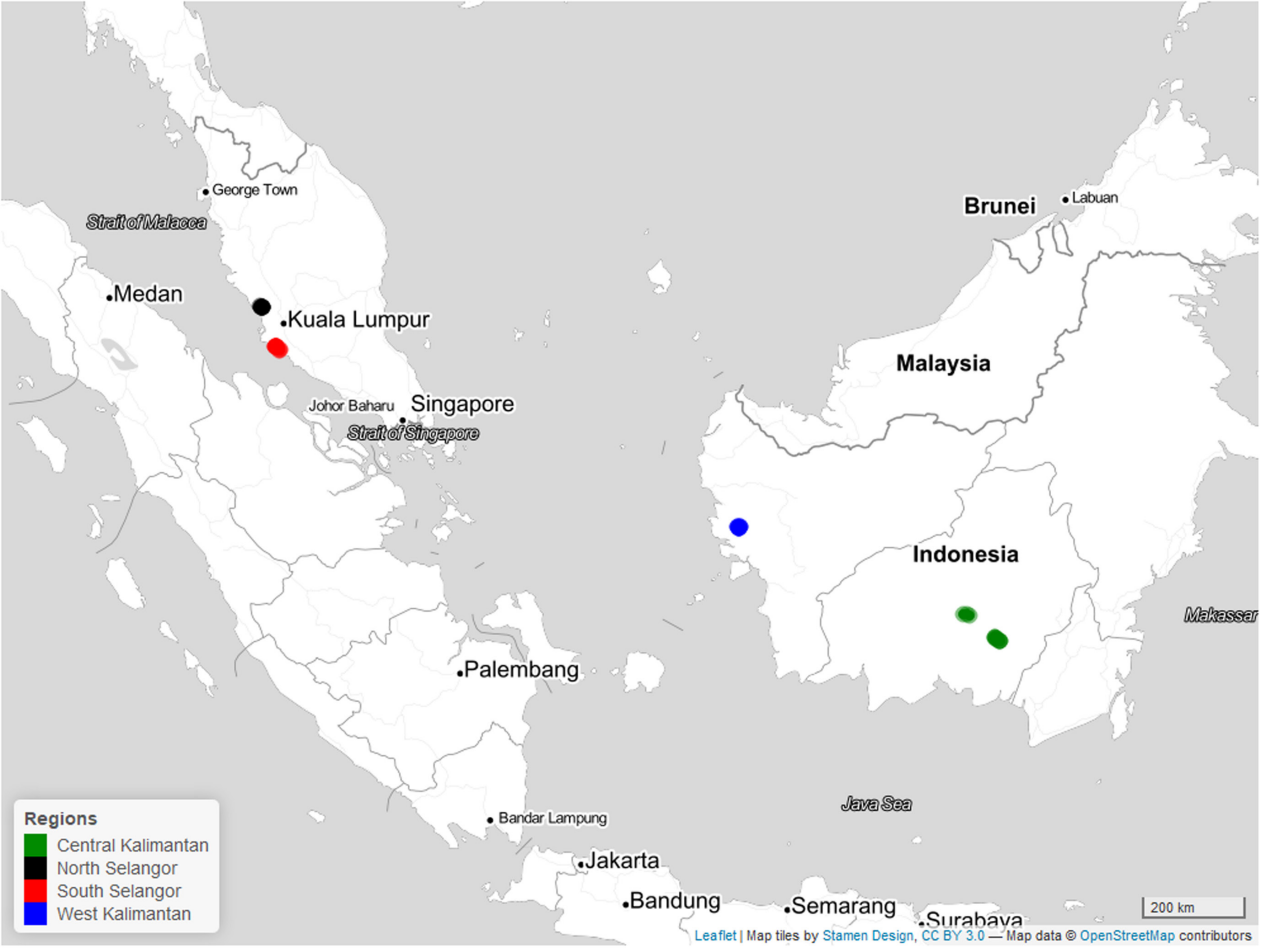
The map displays the locations of the study sites situated in two regions of Peninsular Malaysia and two regions of Kalimantan, Indonesia. Map lines delineate study areas and do not necessarily depict accepted national boundaries.

The study sites in Peninsular Malaysia and in Kalimantan (Indonesian Borneo) are in the humid tropical zone, characterised by heavy rainfall, high temperatures and relative humidity (Figure 1). The Malaysian sites experience two well‐defined seasons, with peaks of rainfall in March–April and in October–November (Selangor State Forestry Department, 2014b). The climate at the Indonesian sites corresponds to an equatorial system (Aldrian & Dwi Susanto, 2003; Kuswanto et al., 2019). The wettest period typically extends from November to April with August being the driest month.

In Peninsular Malaysia, the study sites were located in two regions, North and South of Kuala Lumpur. The North Selangor region was located adjacent to the village of Raja Musa, within the North Selangor PSF, and the South Selangor region was located around the Kuala Langat peatlands complex, southeast of Banting. The North Selangor PSF has experienced logging in the past but is now protected and is subject to rewetting and restoration (Brown et al., 2018; Cooper et al., 2020; Parish et al., 2014). In Kalimantan, the study sites investigated were in the West and Central Kalimantan regions. The study sites in West Kalimantan were located in Teluk Empening, Kubu Raya Regency. Meanwhile, the study sites in Central Kalimantan were located in Kalampangan and Hampangen villages, south and north of Palangka Raya, respectively. The forest sites in Teluk Empening are heavily degraded and have experienced recurring fires. In contrast, the forest sites around Palangka Raya have been rewetted as old unmaintained drainage canals have gradually filled with fallen branches and leaf litter, and this has reduced the water discharge from the sites. Now the forests are in good condition albeit recovering from historical logging. The forest sites have hence all experienced human impacts in some way and show signs of degradation. Their condition ranges from areas with higher water tables and higher biomass to areas with low water tables and low‐standing biomass. We subsequently refer to these forested sites at different stages of degradation as ‘forest’.

In each region, three to four land‐use classes were selected according to local conditions. Land‐use classes fell into four broad classes as classified by IPCC (2014): (a) ‘Forest Land and cleared Forest Land (shrubland), drained’; (b) ‘Plantations, drained, unknown or long rotations’; (c) ‘Plantation, drained, oil palm’; (d) ‘Cropland and fallow, drained’ (Table 1). These land‐use classes are subsequently denoted ‘Forest’, ‘Tree plantation’, ‘Oil palm’ and ‘Cropland’. At each region, four vegetation groups, within the land‐use classes defined above, were selected based on availability of sites. Within each vegetation group, three sites with similar characteristics and land‐use histories were established as replicates (Table S2). Soils at the study sites were classified as deep ombrotrophic lowland peats. Cropland sites in Selangor had shallow peat due to recurring fires, peat subsidence and wastage since agricultural conversion. By contrast, the forest sites had deeper peat. Sites in West Kalimantan had a mix of shallow and deep peat while the Central Kalimantan sites had in general deep peat (Table S2). The organic horizon depth across sites varied between 0.18 and 5.50 m. Annual vegetable crops were grown on raised beds (15–20 cm above ground) of variable lengths and approximately 1 m wide. Tree plantations, which were only present at the Indonesian study sites, were planted at a spacing of approximately 5 × 5 m. Oil palm plantations, of different age classes, were planted in a triangular spacing with distances between palms ranging between 7 to 9.5 m. Farmers at the youngest oil palm sites established an intercropping system consisting of one single harvest of either sweet potato (at two of the sites) or cassava. The short rotation crops (cropland land‐use class) were commonly fertilised close to the stems at the time of planting. No fertilisation of oil palm or tree plantation sites was observed during the sampling period. For more information about the jelutung sites, please see Jaya et al. (2022). Since the tree plantation land‐use class was not available at the Malaysian sites, data were collected from a further short rotation crop in the cropland land‐use class at each of the two Malaysian regions.

**TABLE 1:** Site characteristics summary. Agricultural crops include winter melon (*Benincasa hispida* (Thunb.) Cogn), pineapple (*Ananas comosus* (L.) Merr.), turmeric (*Curcuma longa* L.), banana (BN) (*Musa* sp.), ginger (*Zingiber officinale* Rosc.) and water spinach (*Ipomoea aquatica* Forsk.). Tree plantation includes rubber (*Hevea Brasiliensis* Müll.Arg) and jelutung (*Dyeria costulata* (Miq.) Hook.). Oil palm (*Elaeis guineensis* Jacq.). The subscripted forest site names denote the corrsponding regions, which include North Selangor (NS), South Selangor (SS), West Kalimantan (WK) and Central Kalimantan (CK). Notice that three replicates per vegetation group were selected (see Table S1).

Country	Region	Land‐use class	Vegetation group	Land‐use history^a^	Climatic information^b^
Malaysia	North Selangor	Oil palm	Oil palm	3rd rotation plantation, 16 years old. Shallow drains	Rainfall: 1359–2480
		Cropland	Winter melon	Wasted peat. Shallow drains	Air *T*: 27°C
		Cropland	Pineapple	Wasted peat. Shallow drains	Rel. humidity: 79.3
		Forest	Forest_NS_	Affected by very old drains used to extract timber in the past. Shallow groundwater	
	South Selangor	Oil palm	Oil palm	Former cropland land use. Recently planted. Deep drains	Rainfall: 1359–2480
		Cropland	Turmeric	Wasted peat. Frequent burning and deep drains	Air *T*: 27°C
		Cropland	Banana	Wasted peat. Frequent burning and deep drains	Rel. humidity: 79.3
		Forest	Forest_SS_	Encroached forest with frequent burns on the forest edges	
Indonesia	West Kalimantan	Oil palm	Oil palm	Recently cleared shrubland. 4 years old. Shallow drains	Rainfall: 3091 mm year^−1^
		Tree plantation	Rubber	12 years old plantation. Shallow drains	Air *T*: 26.8°C
		Cropland	Ginger	Recently cleared shrubland. Shallow drains	Rel. humidity: 86%
		Forest	Forest_WK_	Encroached forest, heavily degraded and affected by fire in 2018. No drains within the forest area	
	Central Kalimantan	Oil palm	Oil palm	1st rotation plantation, 9 years old. Deep drains	Rainfall: 2960 mm year^−1^
		Tree plantation	Jelutung	12 years old plantation. Deep drains	Air *T*: 26.9°C
		Cropland	Water spinach	Burned in 2015. Deep drains	Rel. humidity: 83%
		Forest	Forest_CK_	Affected by fire in 1997. Old unmaintained drains. Shallow groundwater	

^a^ Age for the oil palm and tree plantation vegetation groups refers to 2018.

^b^ Data for North Selangor (period 2005–2013) from (Charters et al., 2019); for West and Central Kalimantan (period 1981–2020) from Supadio Airport weather station (Pontianak) and Tjilik Riwut Airport weather station (Palangka Raya), respectively. No specific climatic data for South Selangor.

The main tree species at the North Selangor PSF were *Koompassia malaccensis*, *Shorea uliginosa*, *Xylopia fusca, Santiria* sp. and *Syzygium* sp. (Selangor State Forestry Department, 2014b). Similarly, vegetation at the Kuala Langat South PSF site was dominated by *Shorea teysmanniana*, *Koompassia malaccensis*, *Calophyllum* spp. and *Cratoxylum arborescens*. Forest sites in Indonesia were mostly dominated by two tree species, *Combretocarpus rotundatus* and *Cratoxylum arborescens*. All sites, except the forest locations, were located within 10–100 m of large canals (typically around 1.5 m wide and over 1 m deep). Indonesian forest sites were also affected by surrounding canal systems built along nearby main roads that were between 150 and 500 m from the study sites. Closed canal systems (1 m wide and 1 m deep), that were not intended to have a drainage impact, were built around the forest edge as firebreaks and may have affected the study sites in Malaysia. In addition, agricultural sites had shallower drains (30–40 cm deep) within the farmed area.

Cropland sites had crops planted in rows and used parallel raised bed (or strip) systems to facilitate management of water level, whereas the oil palm and tree plantation sites were planted in grids, at a regular spacing in each case. Therefore, within each replicate site, two sampling plots, at two different locations (close to the crops/stems and between crops/stems), were used to conduct the gas flux measurements. At the cropland sites, the ‘near the crop’ plot was installed within the raised bed or strip cropping area and the other plot was installed within the unplanted furrow between the raised beds. At the oil palm and tree plantation land‐use classes, the ‘near the stem’ plot was established at around 30–50 cm from tree/palm trunks, and the ‘far from the stem’ plot at a point equidistant between the trees/palms. Although the same approach was followed at the forest sites, the ‘far from the stem’ plot was only at around 1.5 m from the nearest tree due to the dense vegetation growing at the study sites. Each plot consisted of a measuring area of 40 × 40 cm in which the exact location for gas flux measurements was marked with four wooden sticks. All ground vegetation (e.g. non‐vascular plants, grasses and tree seedlings) were carefully removed at the beginning of the experiments and on a monthly basis thereafter. Distance between the plots was around 2–3 m at the forest sites and around 1 m at the other sites. Next to each plot, a perforated PVC dip‐well (2 m long) was inserted 1.5 m into the soil. A PVC cap was used to close the dip‐well to prevent ingress of rainfall and other debris. Plots were established between March and May 2018. There were 96 sample plots in total (4 regions × 12 sites × 2 locations).

### 2.2 Gas sampling and ancillary measurements

Gas flux sampling was conducted monthly from March 2018 to February 2019 in Malaysia, and from May 2018 to April 2019 in Indonesia. Gas samples were collected using static chamber methods. Circular chambers made of opaque polypropylene were used. During sampling, the chambers were carefully inserted into the surface of the peat to create a seal against the ground. After the sampling, the chamber was removed to avoid water logging of the surface of the sampling plots, and for the next sampling occasion the same sampling plots were used. The chamber headspace was 11.5 L and the inner diameter was 28 cm. The top of each chamber was perforated with a 15 mm drill bit and a 19 mm rubber Suba‐Seal Septa was inserted through the hole. The joints around the septas were sealed with silicone to prevent gas leakage. Each measurement consisted of four 25 mL gas samples taken at 4 minute intervals through the septa using plastic syringes and hypodermic needles. After each sampling event, 5 mL of gas was flushed out and the remaining 20 mL of sample was injected into 12 mL pre‐evacuated glass vials (Exetainer®; Labco Ltd., UK). Samples were stored at room temperature for up to 6 months prior to analysis. Measurements at the paired plots (near vs. far locations) were conducted in parallel using two chambers, with 1 min delay between the sampling events. Simultaneously with each gas sampling event, air temperature was measured on top of the chamber. Once the gas sampling was concluded, soil temperature was measured vertically at depths of 5 and 10 cm next to each plot. Additionally, WTD was measured inside the dip‐wells using a laser distance measurer and a thin piece of polystyrene, attached to a nylon string, floating on the water surface inside the dip‐well. A negative scale was used to record belowground WTD values and a positive scale for values above the ground surface (i.e. flooding). Thereafter, around 150 mL of water was taken from each dip‐well using a PVC bailer and a measuring jug. The collected water samples were combined for each site and stored in polypropylene bottles of 120 mL (Malaysian sites) and 250 mL (Indonesian sites) capacity. Equipment and bottles were gently rinsed with sample water in triplicate before being filled. Once in the laboratory, water samples were filtered through 0.45 μm cellulose nitrate membrane filters using a vacuum pump. Around 60 mL of filtered water was stored in polypropylene centrifuge tubes and stored in the dark at around 4°C until further analysis. Total dissolved nitrogen (TDN) was measured in a TOC‐V CSH Total Organic Analyser with TNM‐1 Total Nitrogen Measurement unit (Shimadzu, UK). Prior to analysis, 2 mL of water sample was diluted with ultrapure Milli‐Q® water in a 1:10 ratio. A calibration curve containing a blank and five known concentrations of TDN (2, 4, 6, 8 and 10 mg L^−1^) was used to measure TDN concentrations. Average TDN concentration for each site was calculated as the mean value from all months during which water was collected. Mean site TDN values from the same land‐use class were used to calculate an average TDN for each land‐use class.

Gas samples were analysed for CH_4_ and N_2_O using a gas chromatograph (GC; GC‐2014, Shimadzu UK LTD) with two separate channels fitted to a thermal conductivity detector (TCD), a flame ionization detector (FID) and an electron capture detector (ECD) for the determination of CH_4_ and N_2_O concentrations, respectively. The GC was connected to a custom‐made single‐injection auto sampler able to handle up to 56 vials. Each injection consisted of 5 mL of sample flushed into a non‐polar methyl silicone capillary column (CBP1‐W12‐100, 0.53 mm I.D., 12 m, 5 mm; Shimadzu UK LTD) and a porous polymer packed column (HayeSep Q 80/100) using nitrogen as the carrier gas. Sampling in March 2018 at the Malaysian sites was only conducted using Los Gatos. In addition, due to sampling problems, GC data are not available for North and South Selangor between November 2018 and February 2019. Samples collected from the Central Kalimantan sites in April 2019 could not be analysed.

In addition to the static chamber method, a series of intensive gas sampling campaigns (between March–June 2018 and October–January 2019 in both Malaysia and Indonesia) were conducted using a dynamic closed chamber connected to a Los Gatos Ultraportable GHG analyser (San Jose, California, USA). The aim of the Los Gatos data collections was to generate a rapid in situ assessment of the fluxes to understand the rate of gas accumulation in the chamber with time and to detect any potential issues linked to chamber size and measurements at the different type of plots. This was complementary to the vial sampling that was applied in parallel at the four different main study areas. For the Los Gatos measurements, the chamber characteristics were the same as the static chamber except that instead of having a rubber Suba‐Seal Septa on top, this chamber was equipped with two push‐fit connectors that acted as an inlet and outlet allowing the gas to flow from the chamber to the analyser and then back into the chamber. After the chamber was placed on the measuring point, it was gently pushed into a 2 cm deep groove in the peat to create an airtight headspace between the ground surface and the chamber walls. The soil CH_4_ concentration (in ppm) increment inside the chamber was measured during 6–10 min, with data automatically recorded at every 20 s. In addition, real time measurements were streamed on a mobile phone using VNC Viewer and Los Gatos Wi‐Fi capabilities. If the CH_4_ concentration increase over time was considered poor (e.g. plotted data with no apparent linear fit) or if pulses of CH_4_ and / or a gas leakage were detected, the measurement was stopped (i.e. lifting the chamber from the soil) and a new measurement was taken after CH_4_ readings on the mobile phone had stabilised (1 min approximately). As with the static chamber method, once measurements were concluded, soil temperatures at 5 and 10 cm depths and WTD were measured. Around 40% of the soil CH4 fluxes were measured with Los Gatos.

Soil CH_4_ fluxes calculated using Los Gatos and GC analysis were not statistically different (*p* = .76; Figure S1) and therefore, fluxes from both methods were combined to calculate mean hourly and annual fluxes. Further investigation at the plot level showed that, at 3 out of the 84 sampling plots, the CH_4_ fluxes calculated with Los Gatos and GC analysis were significantly different (Figure S2). However, these differences were not consistent across the three sampling plots indicating that no systematic error was linked to the analysis approach.

### 2.3 Data analysis and calculation of emissions factors

Trace gas concentrations, determined by either GC analysis or by Los Gatos, were converted to mass units using the ideal gas law (Equation 1). Thereafter, using the slope of the linear change in gas concentration over time and Equation (2), CH_4_ and N_2_O fluxes were calculated. Flux calculations were automated using R 3.6.3 (R Core Team, 2013) and the *flux* v0.3‐0package (Jurasinski et al., 2014). This package fits a number of linear regressions to the data, retaining a minimum number of concentration values, specified by the user, and then returns the model with the best fit. For fluxes calculated using the static closed chamber method, three out of four values were retained. In the case of fluxes calculated using the dynamic closed chamber, a minimum of 15 concentration values (i.e. 5 min sampling interval) were required to calculate the fluxes. If some flux measurements contained less data points due to system malfunctioning (e.g. flat battery and system overheating), the fluxes were still calculated but a warning message was generated and exported with the data. In this instance, the flux calculation was checked manually to ensure erroneous data was not included in the statistical evaluation of the data. Positive fluxes indicated GHG emissions from the soil into the atmosphere and negative fluxes indicated soil uptake of atmospheric GHG.

(1) $$n=\frac{\mathrm{PV}}{\mathrm{RT}},$$

(2) $$F=\frac{\left(∆n/∆t\right)\times {\text{Volume}}_{\text{chamber}}}{{\text{Area}}_{\text{chamber}}},$$

where, *n* is the number moles of trace gas (mol L^−1^), *P* is the atmospheric pressure (Pa), *V* is the volume of trace gas per litre of air (L L^−1^), *R* is the gas law constant (8314.46 L·Pa·K^−1^·mol^−1^), and *T* is the temperature (K). *F* is the calculated flux for either CH_4_ or N_2_O (mol m^−2^ h^−1^), *∆n/∆t* is the slope of the linear regression (mol L^−1^ h^−1^) (i.e. change in gas concentration inside the chamber over time), Volume_chamber_ is the volume of the chamber headspace (L) and Area_chamber_ is the area of the chamber (m^2^).

Fluxes were converted to μg m^−2^ h^−1^ using the molecular weight of CH_4_ and N_2_O (i.e. 16.04 and 44.01 g mol^−1^, respectively). To avoid excluding very small CH_4_ and N_2_O fluxes from the annual calculations, fluxes with low *r* ^2^ were used in the calculation of cumulative emissions. By contrast, large CH_4_ and N_2_O fluxes with low *r* ^2^ were discarded as these fluxes were considered to be either affected by CH_4_ ebullition, gas leakage or gas under pressured during transportation of vials. As gas CO_2_ concentrations were analysed in parallel with the N_2_O and CH_4_ fluxes (note that this data is not reported in this paper) these fluxes were also used to assess potential problems during sampling. Mean areal values of WTD, CH_4_ and N_2_O fluxes, based on the relative areas occupied by each microtopography, were calculated for each sampling event and site. An equal weight was assigned to the ‘near the stem’ and ‘far from the stem’ locations at the forest, tree plantation and youngest oil palm sites. In contrast, a 30:70 ratio was assigned at the other three oil palm sites, for the near and far locations, respectively. This ratio was derived from Swails et al. (2021). At the cropland sites, the ratio assigned at ‘near the crop’ and ‘far from the crop’ locations were 67:33, respectively, which represented the dimensions of the raised beds and unplanted furrow areas. Site means were calculated as the areal mean value between all sampling events. In addition, annual fluxes were calculated as the site mean × 24 h × 365 days. Furthermore, average CH_4_ and N_2_O emissions for each land‐use class were calculated using the mean values of all sites within each land‐use class, as they were independent from each other. CH_4_ fluxes calculated using Los Gatos and GC analysis techniques were combined together prior to the statistical analysis and to the calculation of the annual fluxes.

### 2.4 Statistical analysis

The relationship between WTD and CH_4_ and N_2_O fluxes was investigated by non‐linear regression analysis. In addition, the relationship between TDN and N_2_O fluxes was tested using linear and non‐linear analysis. Prior to the regression analysis, N_2_O fluxes and TDN concentrations were log‐transformed. A range of model fits to the results (linear, log‐linear, exponential and sigmoidal) were explored and the most appropriate model in each case was selected. Both hourly and mean annual fluxes were used to develop models for a broad range of land‐use classes and environmental conditions. Although not presented here, the effect of soil T10 (as a marginally significant variable) on the GHG fluxes was also investigated, both on its own and in combination with either WTD or TDN, but this resulted in non‐significant models.

After a visual inspection of the datasets, CH_4_ fluxes were fitted to an exponential function with two parameters (Equation 3) and N_2_O fluxes were fitted to a sigmoidal equation with three parameters (Equation 4). In addition, within each land‐use class, a linear regression was fitted between site‐specific annual WTD and N_2_O emissions (Equation 5). These are:

(3) $$\mathrm{flu}x_{{\mathrm{CH}}_{4}}=a_{i}{\left(\exp\right)}^{b_{i}\mathrm{WTD}},$$

(4) $$\mathrm{flu}x_{N_{2}O}=\frac{c_{i}}{1+e^{-\left(\frac{\mathrm{TDN}-{\mathrm{TDN}}_{i}}{d_{i}}\right)}},$$

(5) $$\mathrm{flu}x_{N_{2}O}=m_{i}\mathrm{WTD}+n_{i},$$

where ${\text{flux}}_{{\mathrm{CH}}_{4}}$ and ${\text{flux}}_{N_{2}O}$ are the measured CH_4_ and N_2_O emissions expressed in units of mg m^−2^ h^−1^, WTD is the measured water table depth, *a*_*i*_ and *b*_*i*_ are fitted parameters greater than 0 obtained by non‐linear regression analysis, *c*_*i*_, *d*_*i*_ and WTD_0_ are specific fitted parameters determined using least squares non‐linear regression for each of the equations tested and *m*_*i*_ and *n*_*i*_ are the slope of the regression line and the intercept, respectively.

Statistical analyses were performed using GENSTAT 19 (VSN International, UK). The impacts of the land‐use class, vegetation group (including all annual crops, tree plantation, oil palm and forest sites), location, soil T10 and WTD on CH_4_ and N_2_O fluxes were investigated using mixed linear models, with repeated measurements, using the residual maximum likelihood (REML) method. The effect of TDN on N_2_O fluxes was also tested. In the model, the sampling plots were used as subjects, the site was used as the random effect and the sampling events were used as time points. In the first analysis vegetation group, location (far vs. near the crop/stem), WTD, soil T10 and TDN were the fixed effects in the model. A second model using land‐use class and location (near vs. far) as the fixed effects was also carried out to test for overall differences among the land use classes. The effects of the interactions between the fixed factors on the CH_4_ and N_2_O fluxes were also investigated. As part of the analysis the residual plots were inspected. In instances where the model assumptions were not met data were transformed using either log or BoxCox transformation of CH_4_ and N_2_O fluxes, WTD, soil T10, and TDN before further analysis. Prior to these transformations, variables that had positive and negative values were converted into positive scales only. Standard errors of the differences (SEDs) estimated from the mixed model were used to evaluate which means were different from each other. In addition, statistical differences between paired CH_4_ fluxes, calculated using Los Gatos and GC analysis techniques, were investigated using the non‐parametric Wilcoxon test. For these comparisons, only months for which both techniques were used simultaneously (or within a few days apart) were included. The same statistical non‐parametric method was used to assess potential differences in CH_4_ and N_2_O fluxes measured at the locations (e.g. far vs. near). All non‐linear regression analyses were conducted using SigmaPlot 13 (systat Software Inc. USA) and figures were produced using R (R Core Team, 2013) and the package *ggplot2* (Wickham, 2009). The non‐parametric Wilcoxon test was performed using the R package *ggpubr* (Kassambara, 2021). All the statistical tests were realised at the *p* = .05 significance level.

## 3 RESULTS

### 3.1 CH_4_ and N_2_O fluxes and environmental factors

**FIGURE 2:**
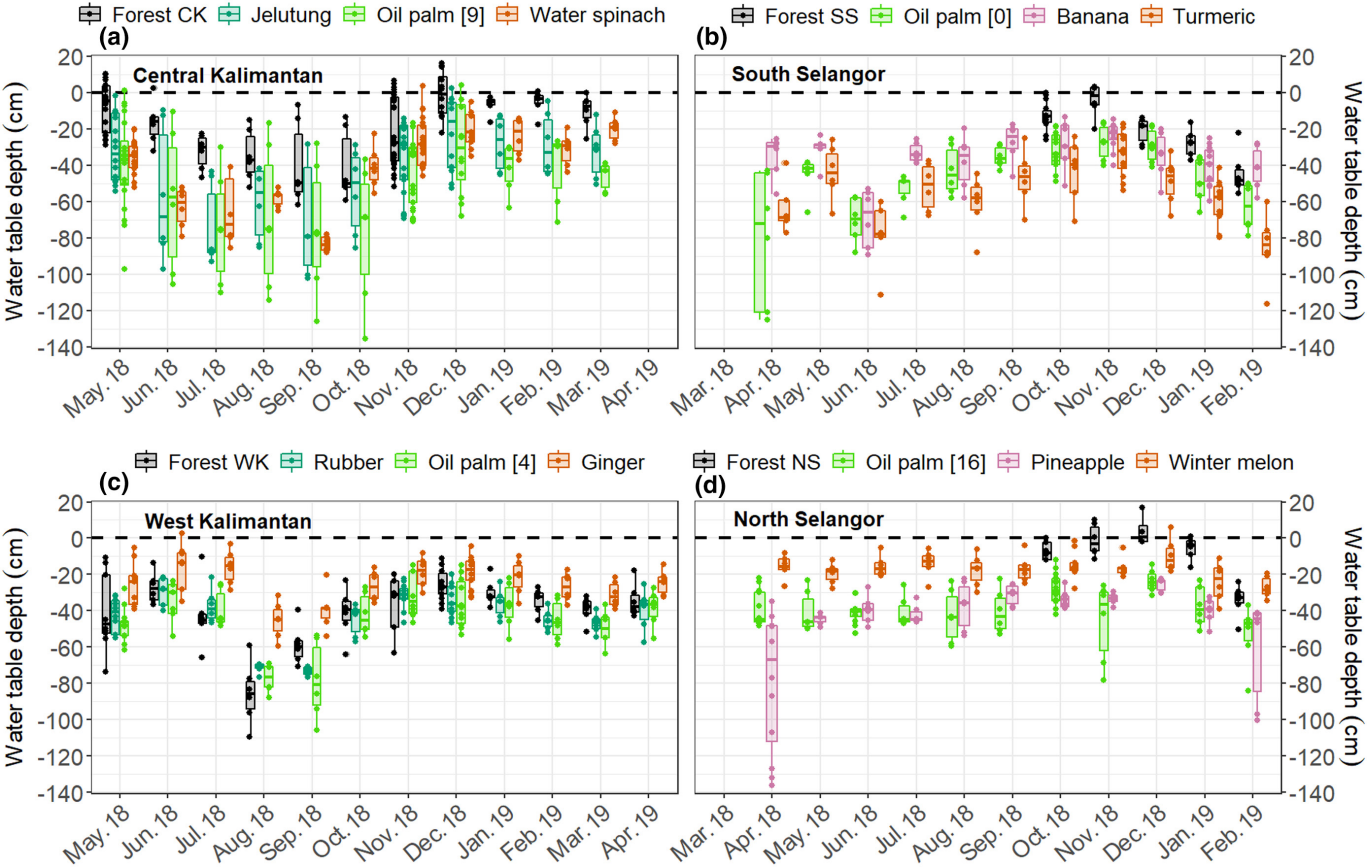
Seasonal variation of water table depth in the four land‐use classes, grey—forest, blue—tree plantations, green—oil palm and orange and pink—cropland. The specific oil palm age, tree plantation species and crop types differed among regions so different plantation and crop types were measured in each location. Note that the forest condition differs substantially among the four regions. Each boxplot represents data from three replicates of each vegetation group.

**FIGURE 3:**
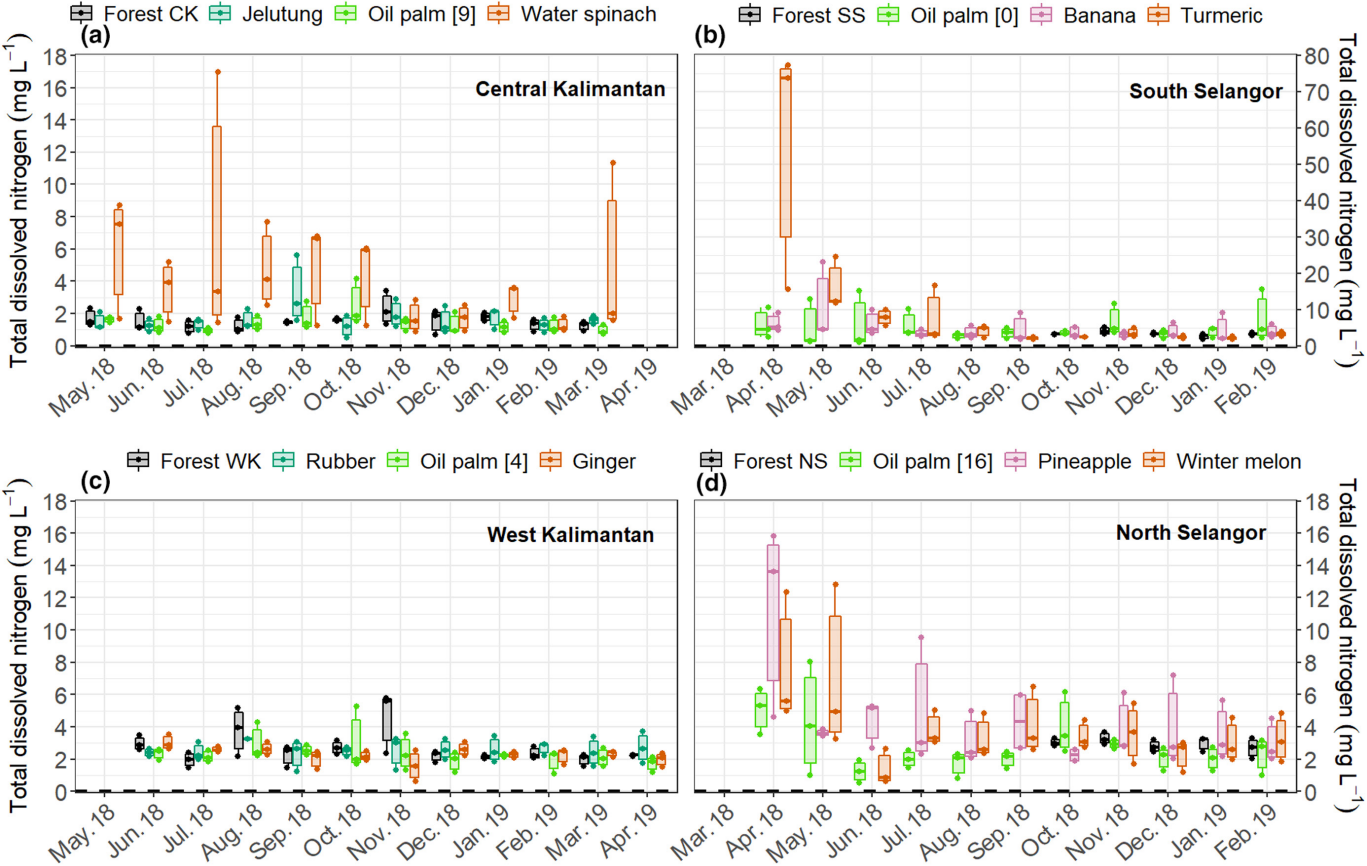
Seasonal variation of total dissolved nitrogen (TDN) in the four land‐use classes, grey—forest, blue—tree plantations, green—oil palm and orange and pink—cropland. The specific oil palm age, tree plantation species and crop types differed among regions so different plantation and crop types were measured in each location. Note that the forest condition differs substantially among the four regions. Each boxplot represents data from three replicates of each vegetation group.

Mean soil temperature was lowest at the forest land‐use class (26.5 ± 0.1°C) and highest at the cropland land‐use class (28.7 ± 0.1°C). Temperatures at these two land‐use classes were significantly lower and higher, respectively, than soil temperatures at the other land‐use classes. By contrast, soil temperatures were similar (between 27.7 ± 0.1 and 27.1 ± 0.1°C) at the oil palm forest and tree plantation land‐use classes (Table S3). Groundwater level had a clear temporal variation related to precipitation. Lowest and highest WTDs were recorded in August–September (usually the driest months) and November–January (usually the wettest months), respectively (Figure 2). Across all study sites, mean WTD varied between −75 ± 6 cm (at the oil palm sites in Central Kalimantan) and −2 ± 2 cm (at the forest sites in Central Kalimantan) (Tables S3 and S4). Although mean WTD was deepest at the oil palm land‐use class (−45 ± 4 cm) it was not significantly different from mean WTD at the tree plantation (−41 ± 6 cm) or cropland (−38 ± 4 cm) land‐use classes. Mean WTD was shallowest at the forest land‐use class (−25 ± 1 cm). Sites from the cropland land‐use class had the highest mean TDN concentrations (Figure 3). The pineapple sites had the greatest mean TDN (37.3 ± 18.0 mg L^−1^), followed by the turmeric sites (17.4 ± 8.0 mg L^−1^), in the North and South Selangor regions, respectively (Table S3). The lowest mean TDN concentration was measured at the oil palm sites in Central Kalimantan (1.1 ± 0.1 mg L^−1^), followed by the forest and jelutung sites in Central Kalimantan (both with 1.1 ± 0.1 mg L^−1^). On average, the cropland and forest land‐use classes had the highest and the lowest mean TDN (7.5 ± 2.0 and 2.1 ± 0.1 mg L^−1^), respectively.

**FIGURE 4:**
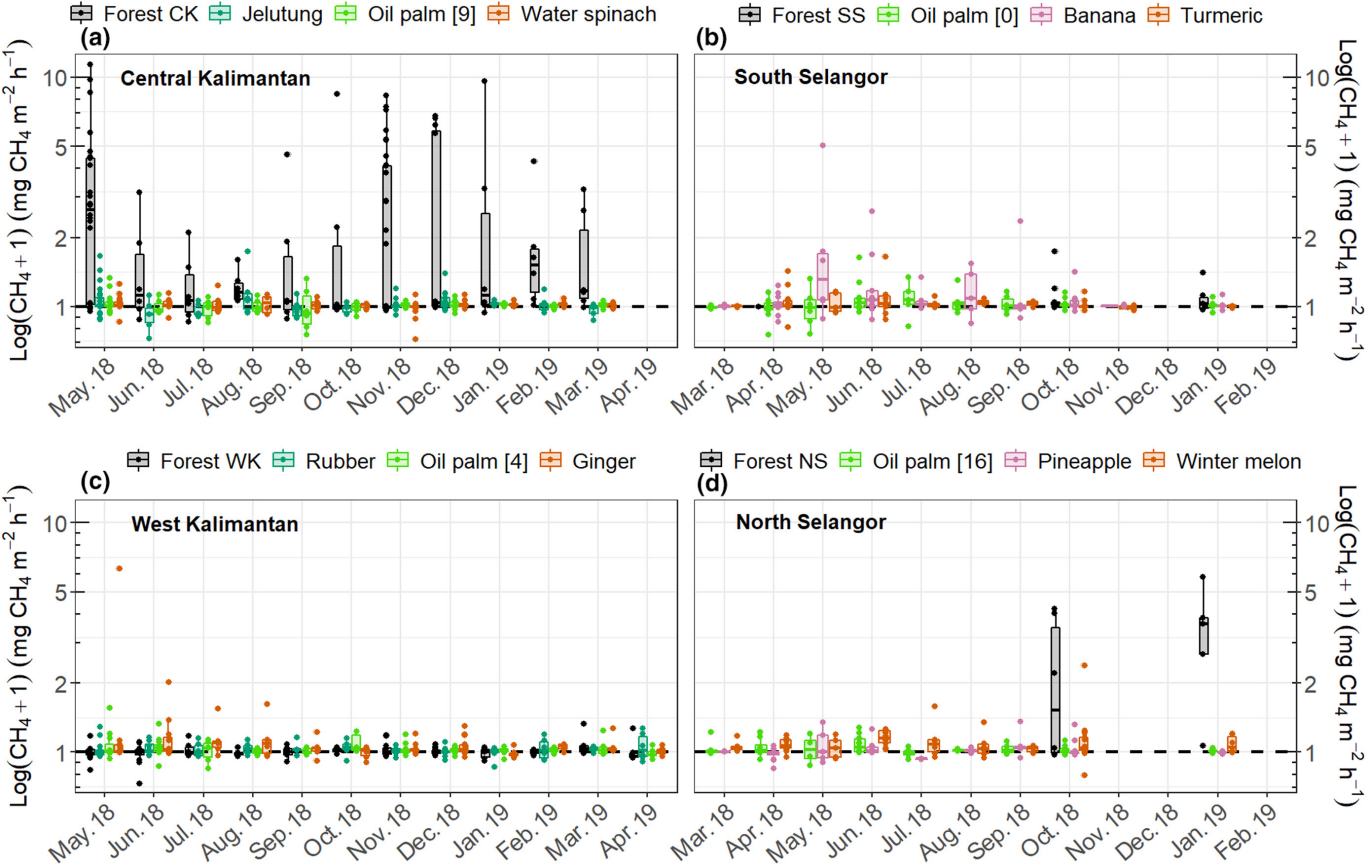
Seasonal variation of hourly CH_4_ fluxes in the four land‐use classes, grey—forest, blue—tree plantations, green—oil palm and orange and pink—cropland. The specific oil palm age, tree plantation species and crop types differed among regions so different plantation and crop types were measured in each location. Note that the forest condition differs substantially among the four regions. Each boxplot represents data from three replicates of each vegetation group. Data from the static and dynamic chambers are combined.

Soil CH_4_ fluxes were generally low across all sites except at the forest sites in Central Kalimantan and North Selangor regions (Figure 4). Mean hourly CH_4_ fluxes varied between −0.05 and 3.60 mg m^−2^ h^−1^ across all study sites (Table S3). Overall, the forest land use class had the highest mean hourly CH_4_ fluxes (0.78 ± 0.1 mg m^−2^ h^−1^). By contrast, the tree plantation and oil palm land‐use classes, which had the deepest WTDs, had the lowest mean hourly CH_4_ fluxes (both with 0.02 ± 0.01 mg m^−2^ h^−1^; Table 2; Tables S3 and S4).

**TABLE 2:** Results from the mixed linear models, with repeated measurements, explaining the variations in CH_4_ and N_2_O fluxes. Fixed terms include vegetation (refers to the specific vegetation groups as described in Table 1), location (near the crop/stem vs. far from the crop/stem where the flux measurements were conducted), T10 (soil temperature at 10 cm depth), WTD (water table depth) and TDN (total dissolved nitrogen). Significant (*p* < .05) and near significant (*p* < .1) effects are shown. The full output table describing also the non‐significant interactions from the mixed model including ‘vegetation group’ as a fixed effect in the analysis are shown in Table S4.

Fixed term	CH_4_ fluxes			N_2_O fluxes				
	Wald statistic	df	Wald/df	Chi pr	Wald statistic	df	Wald/df	Chi pr
**Vegetation**	**35.01**	**13**	**2.69**	**<0.001**	**61.27**	**13**	**4.71**	**<0.001**
**Location**	0.67	1	0.67	ns	**9**	**1**	**9**	**0.003**
*T10*	*3.35*	*1*	*3.35*	*0.067*	2.42	1	2.42	ns
**WTD**	**7.65**	**1**	**7.65**	**0.006**	**12.89**	**1**	**12.89**	**<0.001**
**TDN**	2.39	1	2.39	ns	**17.67**	**1**	**17.67**	**<0.001**
***Vegetation × Location***	*20.37*	*13*	*1.57*	*0.086*	**41.64**	**13**	**3.2**	**<0.001**
**Vegetation *×* T10**	**23.11**	**13**	**1.78**	**0.040**	**52.49**	**13**	**4.04**	**<0.001**
**Vegetation *×* WTD**	**28.44**	**13**	**2.19**	**0.008**	**49.45**	**13**	**3.8**	**<0.001**
**Vegetation *×* TDN**	11.03	13	0.85	ns	**33.57**	**13**	**2.58**	**0.001**
*T10* ***×*** *WTD*	1.31	1	1.31	ns	*2.85*	*1*	*2.85*	*0.092*
*T10* ***×*** *TDN*	0.2	1	0.2	ns	*3.53*	*1*	*3.53*	*0.060*
**Vegetation *×* location *×* WTD**	**23.03**	**13**	**1.77**	**0.041**	**25.68**	**13**	**1.98**	**0.019**
***Vegetation*** ***×** T10* ***×*** *WTD*	*22.16*	*13*	*1.7*	*0.053*	14.76	13	1.14	ns
**Vegetation *×* T10 *×* TDN**	**30.05**	**13**	**2.31**	**0.005**	**34.71**	**13**	**2.67**	**<0.001**
**Location *×* T10 *×* TDN**	0.02	1	0.02	ns	**7.83**	**1**	**7.83**	**0.005**
**Vegetation *×*** T10 ***×*** WTD ***×*** TDN	7.1	13	0.55	ns	**28.73**	**13**	**2.21**	**0.007**
**Land use**	**23.94**	**3**	**7.98**	**<0.001**	**39.87**	**3**	**13.29**	**<0.001**
**Location**	0.06	1	0.06	ns	1.32	1	1.32	ns
**Land use × location**	0.34	3	0.11	ns	2.49	3	0.83	ns

*Note*: Significant differences (*p* < 0.05) are highlighted in bold and marginally significant differences (0.05 < *p* < 0.1) are highlighted in italics.

Abbreviations: df, degrees of freedom; ns, no significant.

Methane and N_2_O fluxes were strongly determined by WTD and vegetation group at a specific site (Table 2). Highest CH_4_ emissions were recorded at the wettest sites in the forest land‐use class. In contrast, the lowest CH_4_ fluxes were found at the driest sites in the oil palm and tree plantations. Fluxes of CH_4_ followed a clear seasonal variation, related to WTD, with highest emissions measured during the wettest months (October to January with another peak in CH_4_ emissions in May) (Figures 2 and 4). In addition, the combination of location (i.e. near vs. far from the crop/stem), WTD and vegetation group had a significant effect on CH_4_ emissions (Table 2; Figure S3).

**FIGURE 5:**
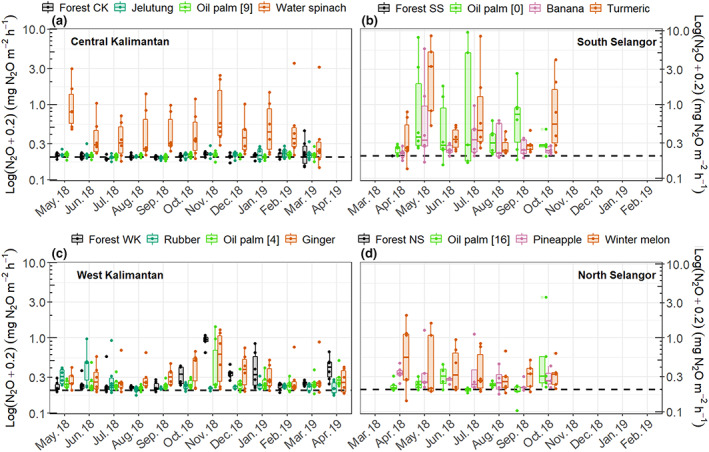
Seasonal variation of hourly N_2_O fluxes in the four land‐use classes, grey—forest, blue—tree plantations, green—oil palm and orange and pink—cropland. The specific oil palm age, tree plantation species and crop types differed among regions so different plantation and crop types were measured in each location. Note that the forest condition differs substantially among the four regions. Each boxplot represents data from three replicates of each vegetation group.

N_2_O fluxes were lowest at the forest and tree plantation sites, and they were greatest at the oil palm and cropland sites (Figure 5; Table 2) with mean hourly N_2_O fluxes varying between <0.001 ± 0.005 and 1.23 ± 0.54 mg m^−2^ h^−1^ across all sites (Table S3). Monthly N_2_O fluxes at the cropland and oil palm land‐use classes followed a seasonal variation, also closely related to WTD, across all regions except at the South Selangor sites where relatively high fluxes were recorded at all sampling events (Figure 5). In addition, the forest site in West Kalimantan followed a similar seasonal pattern to the cropland site, which in West Kalimantan was ginger. The high N_2_O fluxes from the cropland land‐use class corresponded to the soils that had the highest TDN concentrations while the lowest N_2_O fluxes from the forest land‐use class corresponded to the soils with the lowest TDN concentrations (Figure 3).

**FIGURE 6:**
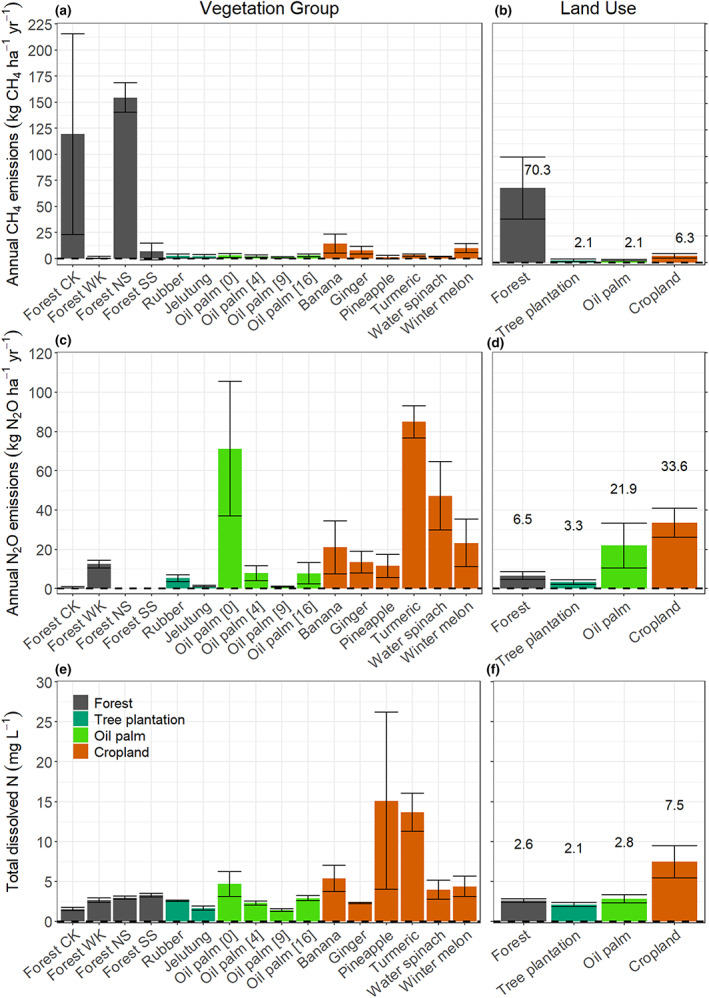
Mean annual CH_4_ and N_2_O emissions by vegetation group (panels a and c) and by land‐use class (panels b and d). Mean total dissolved nitrogen (TDN) by (e) vegetation group and by (f) land‐use class. Each site represents the average of three independent sites. Average CH_4_ and N_2_O emissions for each land use class were calculated using the mean values of all sites within each land use class. Error bars represent standard error of the mean.

Location (i.e. near vs. far from the crop/stem) and TDN had a significant effect on N_2_O emissions (Table 2; Table S4). This effect was more evident at the turmeric, water spinach and recently planted oil palm sites, with higher TDN concentrations (Figure 6e,f) and significantly higher N_2_O fluxes as well. However, responses show some variation among vegetation groups at specific sites over the duration of the experiment (Figure S4).

### 3.2 Annual CH_4_ and N_2_O emission

Almost all study sites were annual CH_4_ sources (Figure 6). On average (±SE), annual CH_4_ emissions (in kg CH_4_ ha^−1^ year^−1^) from the studied land‐use classes were: forest = 70.7 ± 29.5, tree plantation = 2.1 ± 1.2, oil palm plantation = 2.1 ± 0.6 and cropland = 6.2 ± 1.9 (Figure 6b). Similarly, all study sites were N_2_O sources. For the different land‐use classes, annual N_2_O emissions (in kg N_2_O ha^−1^ year^−1^) were: forest = 6.5 ± 2.8, tree plantation = 3.2 ± 1.2, oil palm plantation = 21.9 ± 11.4 and cropland = 33.6 ± 7.3, respectively (Figure 6d).

### 3.3 Modelling of CH_4_ and N_2_O fluxes

Based on the linear mixed model analysis, the effects of WTD and TDN on CH_4_ and N_2_O fluxes were investigated through non‐linear regression analysis.

**FIGURE 7:**
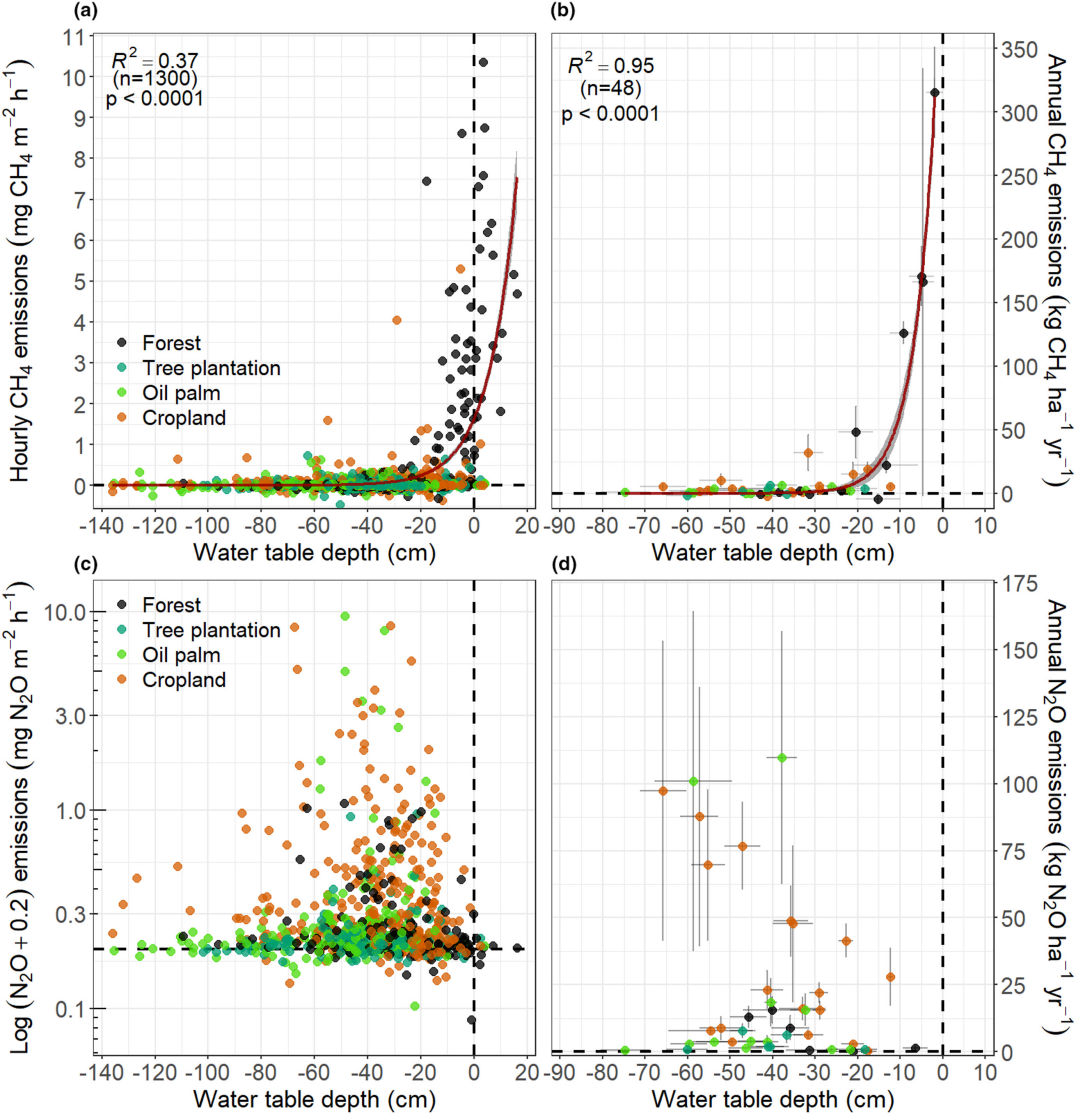
Non‐linear regression analysis between water table depth (WTD) and (a) measured hourly CH_4_ emissions, (b) annual CH_4_ emissions, (c) measured hourly N_2_O emissions and, (d) annual N_2_O emissions. Dataset consists of pooled data from all land use classes and vegetation groups combined. The relationship between the CH_4_ flux and WTD was best explained by an exponential function (Equation 3) in both cases. Error bars correspond to standard error of the mean and shaded area represents the 95% uncertainty of the model.

Methane fluxes were strongly dependent on WTD (Figure 7a,b). Water table depth explained 37% and 95% of the hourly and annual variation in CH_4_ fluxes, respectively (Table 3). At deeper WTDs, CH_4_ fluxes were negligible with some indication of CH_4_ oxidation (i.e. negative CH_4_ fluxes). Methane fluxes increased with higher water levels and net CH_4_ emissions from the soil to the atmosphere started when WTD was approximately −30 and −25 cm, for hourly and annual fluxes respectively (Figure 7a,b).

**TABLE 3:** Model parameters ± standard error of the estimates, *p*‐value of the model (*p*) and coefficients of determinations (*r* ^2^) of non‐linear regression models between CH_4_ flux and water table depth (WTD) through an exponential function (Equation 3) and between N_2_O flux and WTD though a sigmoidal function (Equation 4). Models were developed using data from all land‐use classes and study sites.

Flux	Model	Eq.	Parameters					*p*	*r* ^2^
			*a*_*i*_	*b*_*i*_	*c*_*i*_	*d*_*i*_	TDN_*i*_		
CH_4_	Hourly fluxes	3	1.641 ± 0.060	0.095 ± 0.004	—	—	—	<.0001	.37
	Annual fluxes	3	450.1 ± 25.3	0.195 ± 0.013	—	—	—	<.0001	.95
N_2_O^a^	Annual TDN	4	—	—	1.729 ± 0.241	0.199 ± 0.075	0.400 ± 0.074	<.0001	.47
Significant linear regressions			*m*_*i*_	*n*_*i*_					
N_2_O^a^	Annual Cropland	5	−1.094 ± 0.430	−8.396 ± 17.704	—	—	—	.0001	.29
N_2_O^a^	Annual Forest	5	−0.331 ± 0.158	−2.757 ± 4.936	—	—	—	.051	.52

^a^ Prior to the non‐linear regression analysis, the data were log‐transformed.

**FIGURE 8:**
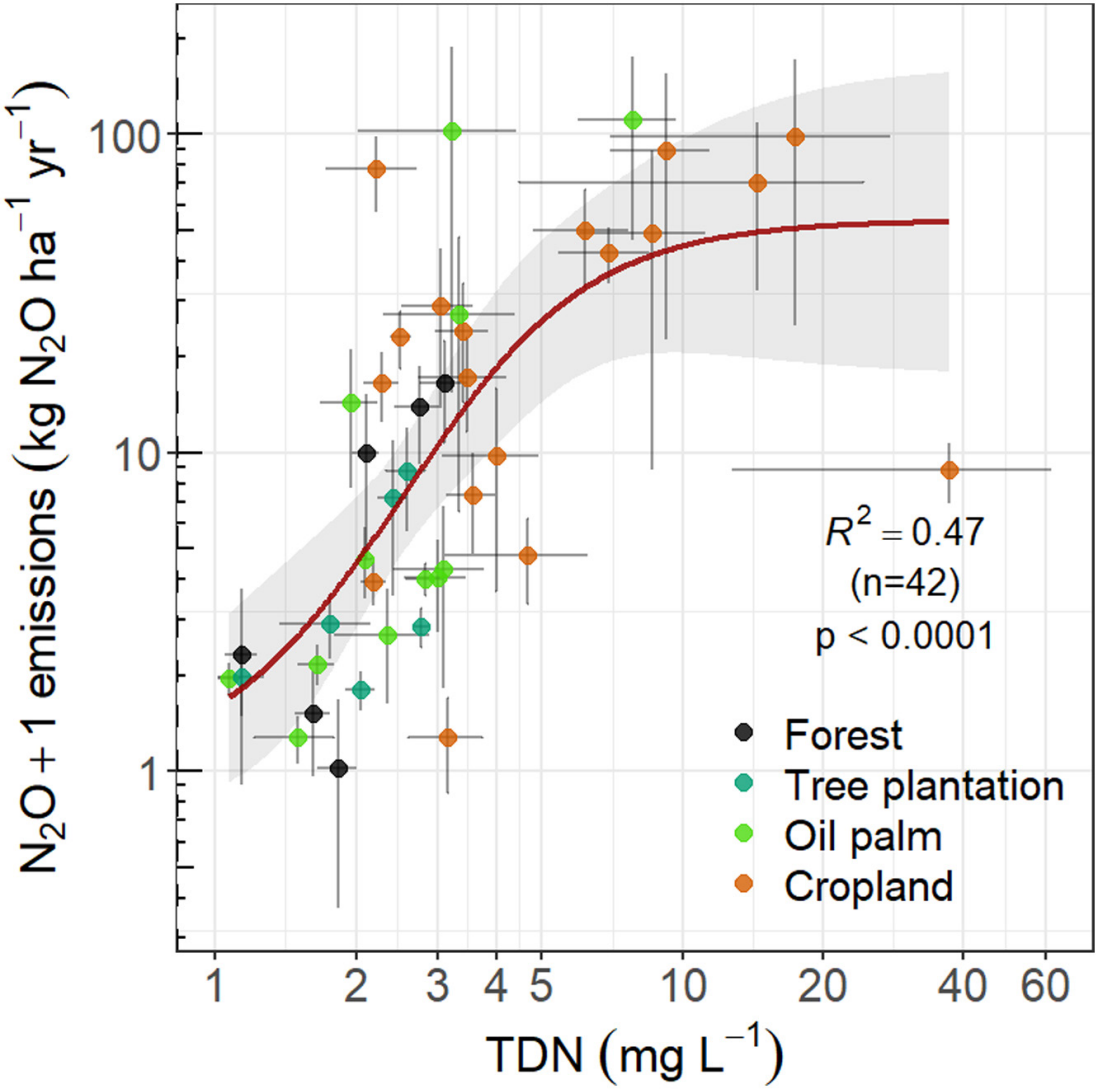
Non‐linear sigmoidal relationship (Equation 4) between mean annual N_2_O fluxes and total dissolved nitrogen (TDN) measured in the phreatic zone. Error bars correspond to standard error of the mean. N_2_O fluxes and TDN concentrations were log‐transformed prior to the regression analysis.

Pooled annual N_2_O fluxes from all land‐use classes and study sites showed that the range in N_2_O emissions was largest when WTD was between ca −20 and −70 cm but did not result in a significant predictive model with WTD (Figure 7c,d). However, cropland‐specific annual N_2_O fluxes had a positive linear relationship with WTD (Figure S5). A marginally significant relationship was found between forest‐specific annual N_2_O emissions and WTD. In both cases, annual N_2_O fluxes increased with deeper WTD, with fluxes close to zero when water level was near the peat surface, and highest during the driest conditions measured (WTD around −40 and −67 cm at the forest and cropland land uses, respectively). Total dissolved nitrogen was positively related to sigmoidal N_2_O emissions (Figure 8). Mean N_2_O fluxes increased with TDN up to a value of around 10 mg L^−1^ but did not increase further where TDN was higher (Table 3). No other significant predictive models were found between CH_4_, N_2_O and the other studied variables.

## 4 DISCUSSION

### 4.1 Environmental drivers of CH_4_ and N_2_O emissions

Soil CH_4_ emissions were strongly dependent on WTD (as also reported by Couwenberg et al., 2010; Deshmukh et al., 2020; Hergoualc'h et al., 2020; Prananto et al., 2020). Hourly CH_4_ fluxes increased exponentially with WTD values higher than ≈ −30 cm. A slightly higher WTD threshold for CH_4_ oxidation/production was found by Jauhiainen et al. (2008) at a PSF affected by drainage and at a deforested and burnt peatland site in Central Kalimantan (i.e. –22 and −12 cm, respectively). Sakabe et al. (2018) found that, in the same PSF in Central Kalimantan, this WTD threshold was −11 cm. Furthermore, similar relationships between WTD and net ecosystem CH_4_ exchange have been found in another recent study, where the production of CH_4_ increased exponentially when WTD was less than ≈ −20 cm (Deshmukh et al., 2020). These results were supported in a meta‐analysis of GHG fluxes from SE Asia, in which a WTD of −20 cm was identified as the threshold for peat soil CH_4_ oxidation/production and deeper values of WTD were associated with very low CH_4_ emissions (Couwenberg et al., 2010). Under flood conditions, methanogenesis dominates biological activity in the soil (see Bridgham et al., 2013 and references therein) and CH_4_ production potential increases with the length of flooding and anaerobic conditions.

The combination of WTD, duration of waterlogging events, together with the quantity and quality of available organic matter, may help explain why approximately 32% of the CH_4_ fluxes used in the empirical modelling (Figure 7a) had WTD values shallower than −30 cm, but CH_4_ fluxes were smaller than 200 μg m^−2^ h^−1^. Flux measurements taken a few days after heavy rain events would have showed a temporarily high WTD but the short duration of these waterlogging events (as a consequence of the drainage systems present at most of the sites) may have prevented the proliferation of methanogens (Sakabe et al., 2018). These combined effects could also help explain why different studies have found different WTD thresholds for soil CH_4_ oxidation/production in tropical peatlands. While WTD thresholds have formerly been suggested for specific land uses (Deshmukh et al., 2020; Jauhiainen et al., 2008; Sakabe et al., 2018), our study has incorporated a broader range of land‐use classes and conditions. A comparable approach by Evans et al. (2021) based on annual CH_4_ fluxes from multiple land‐use classes on temperate peatlands reported a similar WTD threshold (around −30 cm) as the one found in our study. We contend, therefore, that our model, which is based on 48 different sites from several land‐use classes across Malaysia and Indonesia, may provide a robust basis for estimating peat CH_4_ fluxes at both site and regional levels for similar land‐use classes and peatland systems.

Although some incubation experiments have found that under anaerobic conditions, soil temperature has a significant and strong positive exponential effect on soil peat CH_4_ emissions (Sjögersten et al., 2018), no clear effect of soil temperature on CH_4_ emissions has been reported under field experiments on tropical peatlands, probably due to the small variation of temperature in the tropics (Deshmukh et al., 2020; Jauhiainen et al., 2014; Luta et al., 2021). Similarly, we did not find a predictive relationship between in situ temperature and CH_4_ emissions. One possible explanation for these discrepancies between incubation and field experiments could be the low variation in temperature within the forest sites in Central Kalimantan and North Selangor, where the highest CH_4_ fluxes were measured (Deshmukh et al., 2020). For the cropland and young oil palm sites, the temperature range was greater but the deeper WTDs, limited duration of waterlogging events and reduced inputs of fresh litterfall and root exudates resulted in low CH_4_ production (Girkin et al., 2018, 2020; Guillaume et al., 2016; Pulunggono et al., 2019; Van Noordwijk et al., 1997).

Soil N_2_O emissions showed large spatial and temporal variability across all sites but these were in the range reported previously in the region (Hergoualc'h et al., 2020; Oktarita et al., 2017; Swails et al., 2021). Most of this variation was explained by the concentration of TDN in water samples, together with WTD. No data on the amounts of N fertiliser applied by farmers or the N content of the peat soils were obtained. However, TDN is a good indicator of available nitrogen (Hu et al., 2013; Wang et al., 2021) and it could be considered a proxy for the sum of N inputs from fertiliser application and peat mineralisation. Given the much higher TDN concentrations at cropland sites versus drained forest sites with similar WTDs, we infer that differences in TDN were mainly determined by fertiliser application rate. The high TDN concentration found for many of the sampling events at the cropland and oil palm sites suggests local soil treatment practices and in particular, fertiliser application rates with a level of N that exceeds crop demand (Kennedy et al., 2020), are consistent with smallholder agricultural practices reported in Southeast Asia (and elsewhere in Asia) (Poudel et al., 1998; Qiao & Huang, 2021; Zikria & Damayanti, 2019). It is likely that the excess N was available for denitrifying bacteria, a process that produces N_2_O if the denitrification is incomplete (Too et al., 2021; Wolf & Russow, 2000; Xu et al., 2016) before leaching into the groundwater and exiting the peatland complex through the drains and canal systems. Under aerobic and drier conditions, exacerbated by peatland drainage, the mineralisation of organic N to NH_4_^+^ increases and thus, nitrification (conversion of NH_4_^+^ to NO_3_^−^) increases as well. This process can lead to incomplete denitrification and high N_2_O fluxes (Pärn et al., 2018; Rubol et al., 2012), in line with the high N_2_O fluxes with lowered water tables found in our study. Furthermore, our results showed that TDN could explain up to 51% of the variation in N_2_O emissions and that N_2_O emissions rose to a constant value where higher TDN concentrations no longer lead to higher N_2_O emissions (Figure 8). It may be that beyond 10 mg N L^−1^, availability of mineral N is no longer limiting for N_2_O production, with other environmental variables such as soil water content and temperature becoming more important.

### 4.2 Effect of land use on annual CH_4_ and N_2_O emissions

Current IPCC Tier 1 emission factors for smallholder agricultural areas are based on very few empirical data (e.g. one single‐site study for CH_4_ and N_2_O emissions from oil palm, two studies at five sites for CH_4_ from cropland, three studies at eight sites for N_2_O from cropland (IPCC, 2014)). Given the rapid expansion of agriculture on tropical peat, and the potential importance of non‐CO_2_ GHG emissions from these areas, the paucity of data represents a significant evidence gap for national emissions reporting and for the development of effective emissions mitigation measures. The data presented in this study help to address this gap. In particular, our reported CH_4_ and N_2_O fluxes from short rotation agricultural crops could be used to develop regional Tier 1 and country‐specific Tier 2 EFs for this land‐use class and farming system. Furthermore, comparison to other land‐use classes, (i.e. forest, tree plantation and oil palm) constitutes a useful approach to assessing the impacts of different land uses, land‐use change, agricultural management practices and drainage intensity on overall GHG emissions from tropical peatlands.

Annual CH_4_ fluxes from the forests in the present study are within the range of previously reported values for Southeast Asia (Cooper et al., 2020; Deshmukh et al., 2020; Dhandapani, Ritz, Evers, & Sjögersten, 2019; Dhandapani, Ritz, Evers, Yule, et al., 2019; Prananto et al., 2020; Sakabe et al., 2018; Sjögersten et al., 2014). Average CH_4_ emissions at the forest in North Selangor (154 ± 14 kg CH_4_ ha^−1^ year^−1^) were much higher than those previously reported from the same PSF (between −2 and 15 ± 16 kg CH_4_ ha^−1^ year^−1^) (Cooper et al., 2020; Dhandapani, Ritz, Evers, Yule, et al., 2019). However, they were within the range of CH_4_ emissions reported from a ‘good quality dense’ forest in the same area of forest in North Selangor, and from an intact peatland (Terengganu Setiu PSF) located in northeast Peninsular Malaysia (i.e. 438 ± 307 and 175 kg CH_4_ ha^−1^ year^−1^, respectively) (Cooper et al., 2020, Dhandapani, Ritz, Evers, Yule, et al., 2019). The comparatively low range of CH_4_ emissions reported by Dhandapani, Ritz, Evers, Yule, et al. (2019) are a consequence of the drier conditions found at the sampling site, which was affected by several anthropogenic disturbances (e.g. drainage for logging and irrigation of paddy fields, oil palm plantations and paved roads and recently dug 2 m deep ditches). Although our forest sites did not have deep ditches within the forest area, it is likely that the Kuala Langat PSF in South Selangor, was affected by forest encroachment, drainage, forest fires, slash and burn agriculture and oil palm plantations by smallholder farmers and oil palm companies (GEC, 2010; Selangor State Forestry Department, 2014a). These activities had affected the hydrology of the peatland complex and were responsible for the lower CH_4_ emissions measured at the forest sites in South Selangor (between −4 and 22 kg CH_4_ ha^−1^ year^−1^).

Average annual CH_4_ emissions from the forest in Central Kalimantan (i.e. 121 ± 98 kg CH_4_ ha^−1^ year^−1^) were at the higher end of previously reported CH_4_ fluxes from PSF in Kalimantan (between −4 ± 1 and 89 ± 120 kg CH_4_ ha^−1^ year^−1^) (Hirano et al., 2009; Ishikura et al., 2018; Jauhiainen et al., 2008; Sakabe et al., 2018). Our calculated emissions are based on daytime measurements. However, diurnal fluctuation of CH_4_ fluxes (with higher emissions during the day than during the night) have been identified in continuous eddy covariance flux measurements in a PSF in Indonesia (91 ± 9 kg CH_4_ ha^−1^ year^−1^) and Acacia plantation (47 ± 15 kg CH_4_ ha^−1^ year^−1^) (Deshmukh et al., 2020). Although our daytime measurement may overestimate emissions, Deshmukh et al. (2020) reported that this diurnal effect was not significant in a drained Acacia plantation and therefore, we suggest that this would also be the case at the drained tree plantation, oil palm and cropland land uses in our study. Also, the same authors attributed the high CH_4_ emissions at the Acacia plantations to fluxes from the water surfaces of nearby ditches and canals.

During the wet season, CH_4_ emissions from stem fluxes and pneumatophores of some tree species can represent a large fraction of the net ecosystem CH_4_ emissions (Pangala et al., 2013, 2017; Sjögersten et al., 2020). However, our annual CH_4_ fluxes only included peat surface CH_4_ fluxes and therefore, it is possible that the annual CH_4_ fluxes at the forest and tree plantation sites may underestimate total net emissions. Methane production in wetlands is limited by labile organic substrates which are precursors of the substrates needed by methanogens (Bridgham et al., 2013; King et al., 2002; Whiting & Chanton, 1993). Therefore, the regular addition of labile C, nitrogen and phosphorus into the soil from fresh litter and root exudates from the forest vegetation would have also led to higher CH_4_ fluxes by increasing microbial activity and decomposition of recalcitrant organic matter (Girkin et al., 2018; Hoyos‐Santillan et al., 2016; Jauhiainen et al., 2014; Könönen et al., 2016; Sakabe et al., 2018). In contrast, degraded peatlands, with recalcitrant organic matter as a consequence of land‐use conversion (e.g. from degraded forests to cropland), with much drier conditions, changes in vegetation and amount and quality of organic inputs into the soil, can explain the low CH_4_ emissions measured at the other land‐use classes.

N_2_O emissions are released at different rates from both natural tropical peatlands and agricultural land uses (Oktarita et al., 2017; Toma et al., 2011). In this study, N_2_O emissions at the forest land use, were in the lower range of previously reported fluxes in SE Asia (0.3–161.1 kg N_2_O ha^−1^ year^−1^) (Cooper et al., 2020; Ishikura et al., 2018; Jauhiainen et al., 2012). The tree plantation land use had similar annual N_2_O emissions to those reported from degraded forest and tall shrub land‐use classes (1.1–4.5 kg N_2_O ha^−1^ year^−1^) (Hergoualc'h & Verchot, 2014; Murdiyarso et al., 2019; Takakai et al., 2006). The range of reported annual N_2_O emissions from cropland land uses in Southeast Asia varies enormously. While some studies suggest annual fluxes between 0.2 and 2.3 kg N_2_O ha^−1^ year^−1^ (Hergoualc'h & Verchot, 2014; Jauhiainen et al., 2012), another study from Central Kalimantan reported annual N_2_O fluxes between 57 and 306 kg N_2_O ha^−1^ year^−1^ (Takakai et al., 2006). Our mean annual N_2_O flux of 34 ± 7 kg N_2_O ha^−1^ year^−1^ (average of the 18 smallholder cropland sites) lies between these extremes, but with a high variability between sites (Figure 6; Table S3), highlighting the importance of site‐specific variables such as fertiliser application rates and WTD within heterogeneous smallholder farming systems. For example, the linear relationship found between WTD and N_2_O at the cropland and forest land‐use classes supports the hypothesis of Couwenberg et al. (2010) that N_2_O emissions increase with drier conditions. Notwithstanding, it also contrasts with other findings in Southeast Asia which suggest that N_2_O emissions increase when groundwater level is close to the peat surface (Swails et al., 2021). Some other studies assessing N_2_O emissions in different land uses in tropical peatlands in Southeast Asia have not reported clear relationships between WTD and N_2_O fluxes (Ishikura et al., 2018; Jauhiainen et al., 2012; Swails et al., 2021).

This study presents CH_4_ and N_2_O fluxes that would be useful for updating Tier 1 EFs for cropland land use in tropical peatlands. In addition, the development of Tier 2 EFs for this land‐use class in Malaysia and Indonesia would improve the accuracy of country‐wide GHG emissions and inventories. Going forward, one of the main challenges of developing N_2_O EFs is to accurately determine the nitrogen inputs from fertilisers in order to separate soil N_2_O emissions caused by peat oxidation from those caused by applied fertilisers (note that these fluxes are reported separately according to IPCC methodology (IPCC, 2006, 2014)). However, the strong dependence of N_2_O emissions on local agricultural management offers the prospect that a substantial proportion of emissions could be mitigated by improved agronomic and water management. Given that over‐fertilisation of smallholder farming systems represents a significant cost to the farmers and the environment for little or no additional yield benefit (Good & Beatty, 2011; Hendricks et al., 2019; Zhang et al., 2018) and that over‐drainage leads to accelerated soil loss (Evans et al., 2019) and potentially also to reduced crop yields, improved management of smallholder agricultural systems offers the potential both to mitigate GHG emissions and to improve farm incomes and livelihoods.

## CONFLICT OF INTEREST STATEMENT

The authors declare no conflicts of interest.

## Supporting information


Data S1

## Data Availability

The data that support the findings of this study are openly available in the Nottingham Research Data Management Repository at https://doi.org/10.17639/nott.7296.

## References

[gcb16747-bib-0001] Aldrian, E. , & Dwi Susanto, R. (2003). Identification of three dominant rainfall regions within Indonesia and their relationship to sea surface temperature. International Journal of Climatology, 23, 1435–1452. 10.1002/joc.950

[gcb16747-bib-0002] BPS . (2020). Indonesian oil palm statistics 2019. Badan Pusat Statistik (BPS).

[gcb16747-bib-0003] Bridgham, S. D. , Cadillo‐Quiroz, H. , Keller, J. K. , & Zhuang, Q. (2013). Methane emissions from wetlands: Biogeochemical, microbial, and modeling perspectives from local to global scales. Global Change Biology, 19, 1325–1346. 23505021 10.1111/gcb.12131

[gcb16747-bib-0004] Brown, C. , Boyd, D. S. , Sjögersten, S. , Clewley, D. , Evers, S. L. , & Aplin, P. (2018). Tropical peatland vegetation structure and biomass: Optimal exploitation of airborne laser scanning. Remote Sensing, 10, 671.

[gcb16747-bib-0005] Charters, L. J. , Aplin, P. , Marston, C. G. , Padfield, R. , Rengasamy, N. , Bin Dahalan, M. P. , & Evers, S. (2019). Peat swamp forest conservation withstands pervasive land conversion to oil palm plantation in North Selangor, Malaysia. International Journal of Remote Sensing, 40, 7409–7438.

[gcb16747-bib-0006] Cooper, H. V. , Evers, S. , Aplin, P. , Crout, N. , Dahalan, M. P. B. , & Sjogersten, S. (2020). Greenhouse gas emissions resulting from conversion of peat swamp forest to oil palm plantation. Nature Communications, 11, 407. 10.1038/s41467-020-14298-w PMC6972824 31964892

[gcb16747-bib-0007] Couwenberg, J. , Dommain, R. , & Joosten, H. (2010). Greenhouse gas fluxes from tropical peatlands in south‐East Asia. Global Change Biology, 16, 1715–1732.

[gcb16747-bib-0008] Couwenberg, J. , Thiele, A. , Tanneberger, F. , Augustin, J. , Bärisch, S. , Dubovik, D. , Liashchynskaya, N. , Michaelis, D. , Minke, M. , Skuratovich, A. , & Joosten, H. (2011). Assessing greenhouse gas emissions from peatlands using vegetation as a proxy. Hydrobiologia, 674, 67–89.

[gcb16747-bib-0009] Deshmukh, C. S. , Julius, D. , Desai, A. R. , Asyhari, A. , Page, S. E. , Nardi, N. , Susanto, A. P. , Nurholis, N. , Hendrizal, M. , Kurnianto, S. , Suardiwerianto, Y. , Salam, Y. W. , Agus, F. , Astiani, D. , Sabiham, S. , Gauci, V. , & Evans, C. D. (2021). Conservation slows down emission increase from a tropical peatland in Indonesia. Nature Geoscience, 14, 484–490.

[gcb16747-bib-0010] Deshmukh, C. S. , Julius, D. , Evans, C. D. , Nardi , Susanto, A. P. , Page, S. E. , Gauci, V. , Laurén, A. , Sabiham, S. , Agus, F. , Asyhari, A. , Kurnianto, S. , Suardiwerianto, Y. , & Desai, A. R. (2020). Impact of forest plantation on methane emissions from tropical peatland. Global Change Biology, 26, 2477–2495. 10.1111/gcb.15019 31991028 PMC7155032

[gcb16747-bib-0011] Dhandapani, S. , Ritz, K. , Evers, S. , & Sjögersten, S. (2019). GHG emission under different cropping systems in some Histosols of Malaysia. Geoderma Regional, 18, e00229.

[gcb16747-bib-0012] Dhandapani, S. , Ritz, K. , Evers, S. , Yule, C. M. , & Sjögersten, S. (2019). Are secondary forests second‐rate? Comparing peatland greenhouse gas emissions, chemical and microbial community properties between primary and secondary forests in peninsular Malaysia. Science of the Total Environment, 655, 220–231. 30471590 10.1016/j.scitotenv.2018.11.046

[gcb16747-bib-0013] Euler, M. , Schwarze, S. , Siregar, H. , & Qaim, M. (2016). Oil palm expansion among smallholder farmers in Sumatra, Indonesia. Journal of Agricultural Economics, 67, 658–676.

[gcb16747-bib-0014] Evans, C. D. , Peacock, M. , Baird, A. J. , Artz, R. R. E. , Burden, A. , Callaghan, N. , Chapman, P. J. , Cooper, H. M. , Coyle, M. , Craig, E. , Cumming, A. , Dixon, S. , Gauci, V. , Grayson, R. P. , Helfter, C. , Heppell, C. M. , Holden, J. , Jones, D. L. , Kaduk, J. , … Morrison, R. (2021). Overriding water table control on managed peatland greenhouse gas emissions. Nature, 593, 548–552. 33882562 10.1038/s41586-021-03523-1

[gcb16747-bib-0015] Evans, C. D. , Williamson, J. M. , Kacaribu, F. , Irawan, D. , Suardiwerianto, Y. , Hidayat, M. F. , Laurén, A. , & Page, S. E. (2019). Rates and spatial variability of peat subsidence in acacia plantation and forest landscapes in Sumatra, Indonesia. Geoderma, 338, 410–421.

[gcb16747-bib-0016] Evers, S. , Yule, C. M. , Padfield, R. , O'reilly, P. , & Varkkey, H. (2017). Keep wetlands wet: The myth of sustainable development of tropical peatlands—Implications for policies and management. Global Change Biology, 23, 534–549. 27399889 10.1111/gcb.13422

[gcb16747-bib-0017] GEC . (2010). Rapid assessment of of Kuala Langat South Peat Swamp Forest in Selangor .

[gcb16747-bib-0018] Girkin, N. T. , Dhandapani, S. , Evers, S. , Ostle, N. , Turner, B. L. , & Sjögersten, S. (2020). Interactions between labile carbon, temperature and land use regulate carbon dioxide and methane production in tropical peat. Biogeochemistry, 147, 87–97. 10.1007/s10533-019-00632-y

[gcb16747-bib-0019] Girkin, N. T. , Turner, B. L. , Ostle, N. , Craigon, J. , & Sjögersten, S. (2018). Root exudate analogues accelerate CO_2_ and CH_4_ production in tropical peat. Soil Biology and Biochemistry, 117, 48–55.

[gcb16747-bib-0020] Good, A. G. , & Beatty, P. H. (2011). Fertilizing nature: A tragedy of excess in the commons. PLoS Biology, 9, e1001124. 21857803 10.1371/journal.pbio.1001124 PMC3156687

[gcb16747-bib-0021] Guillaume, T. , Holtkamp, A. M. , Damris, M. , Brümmer, B. , & Kuzyakov, Y. (2016). Soil degradation in oil palm and rubber plantations under land resource scarcity. Agriculture, Ecosystems & Environment, 232, 110–118.

[gcb16747-bib-0022] Hadi, A. , Inubushi, K. , Furukawa, Y. , Purnomo, E. , Rasmadi, M. , & Tsuruta, H. (2005). Greenhouse gas emissions from tropical peatlands of Kalimantan, Indonesia. Nutrient Cycling in Agroecosystems, 71, 73–80.

[gcb16747-bib-0023] Hendricks, G. S. , Shukla, S. , Roka, F. M. , Sishodia, R. P. , Obreza, T. A. , Hochmuth, G. J. , & Colee, J. (2019). Economic and environmental consequences of overfertilization under extreme weather conditions. Journal of Soil and Water Conservation, 74, 160–171.

[gcb16747-bib-0024] Hergoualc'h, K. , Dezzeo, N. , Verchot, L. V. , Martius, C. , Van Lent, J. , Del Aguila‐Pasquel, J. , & López Gonzales, M. (2020). Spatial and temporal variability of soil N_2_O and CH_4_ fluxes along a degradation gradient in a palm swamp peat forest in the Peruvian Amazon. Global Change Biology, 26, 7198–7216. 32949077 10.1111/gcb.15354 PMC7756671

[gcb16747-bib-0025] Hergoualc'h, K. , & Verchot, L. V. (2014). Greenhouse gas emission factors for land use and land‐use change in southeast Asian peatlands. Mitigation and Adaptation Strategies for Global Change, 19, 789–807.

[gcb16747-bib-0026] Hirano, T. , Jauhiainen, J. , Inoue, T. , & Takahashi, H. (2009). Controls on the carbon balance of tropical peatlands. Ecosystems, 12, 873–887.

[gcb16747-bib-0027] Hoyos‐Santillan, J. , Lomax, B. H. , Large, D. , Turner, B. L. , Boom, A. , Lopez, O. R. , & Sjögersten, S. (2016). Quality not quantity: Organic matter composition controls of CO_2_ and CH_4_ fluxes in neotropical peat profiles. Soil Biology and Biochemistry, 103, 86–96.

[gcb16747-bib-0028] Hu, Z. , Lee, J. W. , Chandran, K. , Kim, S. , Sharma, K. , Brotto, A. C. , & Khanal, S. K. (2013). Nitrogen transformations in intensive aquaculture system and its implication to climate change through nitrous oxide emission. Bioresource Technology, 130, 314–320. 10.1016/j.biortech.2012.12.033 23313675

[gcb16747-bib-0029] Inubushi, K. , Furukawa, Y. , Hadi, A. , Purnomo, E. , & Tsuruta, H. (2003). Seasonal changes of CO_2_, CH_4_ and N_2_O fluxes in relation to land‐use change in tropical peatlands located in coastal area of South Kalimantan. Chemosphere, 52, 603–608. 12738298 10.1016/S0045-6535(03)00242-X

[gcb16747-bib-0030] IPCC . (2006). IPCC guidelines for national greenhouse gas inventories . http://www.ipcc‐nggip.iges.or.jp/public/2006gl/vol1.html

[gcb16747-bib-0031] IPCC . (2014). 2013 supplement to the 2006 IPCC guidelines for national greenhouse gas inventories: Wetlands. Hiraishi, T., Krug, T., Tanabe, K., Srivastava, N., Baasansuren, J., Fukuda, M., Troxler, T.G. (Eds.). IPCC.

[gcb16747-bib-0032] Ishikura, K. , Darung, U. , Inoue, T. , & Hatano, R. (2018). Variation in soil properties regulate greenhouse gas fluxes and global warming potential in three land use types on tropical peat. Atmosphere, 9, 465.

[gcb16747-bib-0033] Jauhiainen, J. , Kerojoki, O. , Silvennoinen, H. , Limin, S. , & Vasander, H. (2014). Heterotrophic respiration in drained tropical peat is greatly affected by temperature—A passive ecosystem cooling experiment. Environmental Research Letters, 9, 105013.

[gcb16747-bib-0034] Jauhiainen, J. , Limin, S. , Silvennoinen, H. , & Vasander, H. (2008). Carbon dioxide and methane fluxes in drained tropical peat before and after hhydrological restoration. Ecology, 89, 3503–3514. 19137955 10.1890/07-2038.1

[gcb16747-bib-0035] Jauhiainen, J. , Page, S. E. , & Vasander, H. (2016). Greenhouse gas dynamics in degraded and restored tropical peatlands. Mires and Peat, 17, 1–12.

[gcb16747-bib-0036] Jauhiainen, J. , Silvennoinen, H. , Hämäläinen, R. , Kusin, K. , Limin, S. , Raison, R. J. , & Vasander, H. (2012). Nitrous oxide fluxes from tropical peat with different disturbance history and management. Biogeosciences, 9, 1337–1350.

[gcb16747-bib-0037] Jaya, A. , Elia, A. , Antang, E. U. , Octora, M. , Ichriani, G. I. , Dohong, S. , & Sulistiyanto, Y. (2022). A study of agroforestry farming for tropical peatland conservation and rehabilitation in Central Kalimantan, Indonesia. Mires and Peat, 28, 1–34.

[gcb16747-bib-0038] Jelsma, I. , Schoneveld, G. C. , Zoomers, A. , & Van Westen, A. C. M. (2017). Unpacking Indonesia's independent oil palm smallholders: An actor‐disaggregated approach to identifying environmental and social performance challenges. Land Use Policy, 69, 281–297. 10.1016/j.landusepol.2017.08.012

[gcb16747-bib-0039] Jurasinski, G. , Koebsch, F. , Guenther, A. , Beetz, S. , & Jurasinski, M. G. (2014). Package ‘flux’. *Flux rate calculation from dynamic closed chamber measurement: R* .

[gcb16747-bib-0040] Kassambara, A. (2021). ggpubr:‘ggplot2’ based publication ready plots . (R package Version 0.4. 0, 2020).

[gcb16747-bib-0041] Kennedy, C. D. , Buda, A. R. , & Bryant, R. B. (2020). Amounts, forms, and management of nitrogen and phosphorus export from agricultural peatlands. Hydrological Processes, 34, 1768–1781.

[gcb16747-bib-0042] King, J. Y. , Reeburgh, W. S. , Thieler, K. K. , Kling, G. W. , Loya, W. M. , Johnson, L. C. , & Nadelhoffer, K. J. (2002). Pulse‐labeling studies of carbon cycling in Arctic tundra ecosystems: The contribution of photosynthates to methane emission. Global Biogeochemical Cycles, 16, 10‐1‐10‐8. 10.1029/2001GB001456

[gcb16747-bib-0043] Könönen, M. , Jauhiainen, J. , Laiho, R. , Spetz, P. , Kusin, K. , Limin, S. , & Vasander, H. (2016). Land use increases the recalcitrance of tropical peat. Wetlands Ecology and Management, 24, 717–731.

[gcb16747-bib-0044] Kuswanto, H. , Setiawan, D. , & Sopaheluwakan, A. (2019). Clustering of precipitation pattern in Indonesia using TRMM satellite data. Engineering, Technology & Applied Science Research, 9, 4484–4489.

[gcb16747-bib-0045] Leifeld, J. , Steffens, M. , & Galego‐Sala, A. (2012). Sensitivity of peatland carbon loss to organic matter quality. Geophysical Research Letters, 39. 10.1029/2012GL051856

[gcb16747-bib-0046] Luta, W. , Ahmed, O. H. , Omar, L. , Heng, R. K. J. , Choo, L. N. L. K. , Jalloh, M. B. , Musah, A. A. , & Abdu, A. (2021). Water table fluctuation and methane emission in pineapples (*Ananas comosus* (L.) Merr.) cultivated on a tropical peatland. Agronomy, 11, 1448.

[gcb16747-bib-0047] Matysek, M. , Evers, S. , Samuel, M. K. , & Sjogersten, S. (2018). High heterotrophic CO_2_ emissions from a Malaysian oil palm plantations during dry‐season. Wetlands Ecology and Management, 26, 415–424.

[gcb16747-bib-0048] McCalmont, J. , Kho, L. K. , Teh, Y. A. , Lewis, K. , Chocholek, M. , Rumpang, E. , & Hill, T. (2021). Short‐ and long‐term carbon emissions from oil palm plantations converted from logged tropical peat swamp forest. Global Change Biology, 27, 2361–2376. 33528067 10.1111/gcb.15544

[gcb16747-bib-0049] Miettinen, J. , Hooijer, A. , Vernimmen, R. , Liew, S. C. , & Page, S. E. (2017). From carbon sink to carbon source: Extensive peat oxidation in insular Southeast Asia since 1990. Environmental Research Letters, 12, 024014.

[gcb16747-bib-0050] Miettinen, J. , Shi, C. , & Liew, S. C. (2016). Land cover distribution in the peatlands of peninsular Malaysia, Sumatra and Borneo in 2015 with changes since 1990. Global Ecology and Conservation, 6, 67–78. 10.1016/j.gecco.2016.02.004

[gcb16747-bib-0051] Mishra, S. , Page, S. E. , Cobb, A. R. , Lee, J. S. H. , Jovani‐Sancho, A. J. , Sjögersten, S. , Jaya, A. , Aswandi , & Wardle, D. A. (2021). Degradation of southeast Asian tropical peatlands and integrated strategies for their better management and restoration. Journal of Applied Ecology, 58, 1370–1387. 10.1111/1365-2664.13905

[gcb16747-bib-0052] Murdiyarso, D. , Saragi‐Sasmito, M. F. , & Rustini, A. (2019). Greenhouse gas emissions in restored secondary tropical peat swamp forests. Mitigation and Adaptation Strategies for Global Change, 24, 507–520.

[gcb16747-bib-0053] Oktarita, S. , Hergoualc'h, K. , Anwar, S. , & Verchot, L. V. (2017). Substantial N_2_O emissions from peat decomposition and N fertilization in an oil palm plantation exacerbated by hotspots. Environmental Research Letters, 12, 104007.

[gcb16747-bib-0054] Page, S. , Mishra, S. , Agus, F. , Anshari, G. , Dargie, G. , Evers, S. , Jauhiainen, J. , Jaya, A. , Jovani‐Sancho, A. J. , Laurén, A. , Sjögersten, S. , Suspense, I. A. , Wijedasa, L. S. , & Evans, C. D. (2022). Anthropogenic impacts on lowland tropical peatland biogeochemistry. Nature Reviews Earth & Environment, 3, 426–443.

[gcb16747-bib-0056] Pangala, S. R. , Enrich‐Prast, A. , Basso, L. S. , Peixoto, R. B. , Bastviken, D. , Hornibrook, E. R. C. , Gatti, L. V. , Marotta, H. , Calazans, L. S. B. , Sakuragui, C. M. , Bastos, W. R. , Malm, O. , Gloor, E. , Miller, J. B. , & Gauci, V. (2017). Large emissions from floodplain trees close the Amazon methane budget. Nature, 552, 230–234. 29211724 10.1038/nature24639

[gcb16747-bib-0057] Pangala, S. R. , Moore, S. , Hornibrook, E. R. C. , & Gauci, V. (2013). Trees are major conduits for methane egress from tropical forested wetlands. New Phytologist, 197, 524–531. 23253335 10.1111/nph.12031

[gcb16747-bib-0058] Parish, F. , Dahalan, M. , & Rahim, H. (2014). Integrated management plan for north Selangor peat swamp forest 2014–2023 for Selangor State Forestry Department . Draft (30 June 2014) Revision, 2.

[gcb16747-bib-0059] Pärn, J. , Verhoeven, J. T. A. , Butterbach‐Bahl, K. , Dise, N. B. , Ullah, S. , Aasa, A. , Egorov, S. , Espenberg, M. , Järveoja, J. , Jauhiainen, J. , Kasak, K. , Klemedtsson, L. , Kull, A. , Laggoun‐Défarge, F. , Lapshina, E. D. , Lohila, A. , Lõhmus, K. , Maddison, M. , Mitsch, W. J. , … Mander, Ü. (2018). Nitrogen‐rich organic soils under warm well‐drained conditions are global nitrous oxide emission hotspots. Nature Communications, 9, 1135. 10.1038/s41467-018-03540-1 PMC5859301 29555906

[gcb16747-bib-0060] Poudel, D. D. , Midmore, D. J. , & Hargrove, W. L. (1998). An analysis of commercial vegetable farms in relation to sustainability in the uplands of Southeast Asia. Agricultural Systems, 58, 107–128.

[gcb16747-bib-0061] Prananto, J. A. , Minasny, B. , Comeau, L.‐P. , Rudiyanto, R. , & Grace, P. (2020). Drainage increases CO_2_ and N_2_O emissions from tropical peat soils. Global Change Biology, 26, 4583–4600. 32391633 10.1111/gcb.15147

[gcb16747-bib-0062] Pulunggono, H. B. , Anwar, S. , Mulyanto, B. , & Sabiham, S. (2019). Decomposition of oil palm frond and leaflet residues. Agrivita, 2019(41), 13.

[gcb16747-bib-0063] Qiao, F.‐B. , & Huang, J.‐K. (2021). Farmers' risk preference and fertilizer use. Journal of Integrative Agriculture, 20, 1987–1995.

[gcb16747-bib-0064] R Core Team . (2013). R: A language and environment for statistical computing. R Foundation for Statistical Computing.

[gcb16747-bib-0065] Rubol, S. , Silver, W. L. , & Bellin, A. (2012). Hydrologic control on redox and nitrogen dynamics in a peatland soil. Science of the Total Environment, 432, 37–46. 22705904 10.1016/j.scitotenv.2012.05.073

[gcb16747-bib-0066] Sakabe, A. , Itoh, M. , Hirano, T. , & Kusin, K. (2018). Ecosystem‐scale methane flux in tropical peat swamp forest in Indonesia. Global Change Biology, 24, 5123–5136. 30175421 10.1111/gcb.14410

[gcb16747-bib-0067] Selangor State Forestry Department . (2014a). Blueprint for Kuala Langat south Forest reserve, Selangor .

[gcb16747-bib-0068] Selangor State Forestry Department . (2014b). Integrated management plan for North Selangor peat swamp forest 2014–2023 . Volume 1, Main plan.

[gcb16747-bib-0069] Sjögersten, S. , Aplin, P. , Gauci, V. , Peacock, M. , Siegenthaler, A. , & Turner, B. L. (2018). Temperature response of ex‐situ greenhouse gas emissions from tropical peatlands: Interactions between forest type and peat moisture conditions. Geoderma, 324, 47–55.

[gcb16747-bib-0070] Sjögersten, S. , Black, C. R. , Evers, S. , Hoyos‐Santillan, J. , Wright, E. L. , & Turner, B. L. (2014). Tropical wetlands: A missing link in the global carbon cycle? Global Biogeochemical Cycles, 28, 1371–1386. 10.1002/2014GB004844 26074666 PMC4461074

[gcb16747-bib-0071] Sjögersten, S. , Siegenthaler, A. , Lopez, O. R. , Aplin, P. , Turner, B. , & Gauci, V. (2020). Methane emissions from tree stems in neotropical peatlands. New Phytologist, 225, 769–781. 31495939 10.1111/nph.16178 PMC6973267

[gcb16747-bib-0072] Swails, E. , Hergoualc'h, K. , Verchot, L. , Novita, N. , & Lawrence, D. (2021). Spatio‐temporal variability of peat CH_4_ and N_2_O fluxes and their contribution to peat GHG budgets in Indonesian forests and oil palm plantations. Frontiers in Environmental Science, 9. 10.3389/fenvs.2021.617828

[gcb16747-bib-0073] Takakai, F. , Morishita, T. , Hashidoko, Y. , Darung, U. , Kuramochi, K. , Dohong, S. , Limin, S. H. , & Hatano, R. (2006). Effects of agricultural land‐use change and forest fire on N_2_O emission from tropical peatlands, Central Kalimantan, Indonesia. Soil Science & Plant Nutrition, 52, 662–674.

[gcb16747-bib-0074] Toma, Y. , Takakai, F. , Darung, U. , Kuramochi, K. , Limin, S. H. , Dohong, S. , & Hatano, R. (2011). Nitrous oxide emission derived from soil organic matter decomposition from tropical agricultural peat soil in Central Kalimantan, Indonesia. Soil Science and Plant Nutrition, 57, 436–451.

[gcb16747-bib-0075] Too, C. C. , Ong, K. S. , Yule, C. M. , & Keller, A. (2021). Putative roles of bacteria in the carbon and nitrogen cycles in a tropical peat swamp forest. Basic and Applied Ecology, 52, 109–123.

[gcb16747-bib-0076] Van Noordwijk, M. , Cerri, C. , Woomer, P. L. , Nugroho, K. , & Bernoux, M. (1997). Soil carbon dynamics in the humid tropical forest zone. Geoderma, 79, 187–225.

[gcb16747-bib-0077] Wang, L. , Xin, J. , Nai, H. , & Zheng, X. (2021). Effects of different fertilizer applications on nitrogen leaching losses and the response in soil microbial community structure. Environmental Technology and Innovation, 23, 101608. 10.1016/j.eti.2021.101608

[gcb16747-bib-0078] Whiting, G. J. , & Chanton, J. P. (1993). Primary production control of methane emission from wetlands. Nature, 364, 794–795.

[gcb16747-bib-0079] Wickham, H. (2009). ggplot2: Elegant graphics for data analysis (use R!). Springer.

[gcb16747-bib-0080] Wijedasa, L. S. , Sloan, S. , Page, S. E. , Clements, G. R. , Lupascu, M. , & Evans, T. A. (2018). Carbon emissions from south‐east Asian peatlands will increase despite emission‐reduction schemes. Global Change Biology, 24, 4598–4613. 29855120 10.1111/gcb.14340

[gcb16747-bib-0081] Wolf, I. , & Russow, R. (2000). Different pathways of formation of N_2_O, N_2_ and NO in black earth soil. Soil Biology and Biochemistry, 32, 229–239.

[gcb16747-bib-0082] Xu, X. , Ran, Y. , Li, Y. , Zhang, Q. , Liu, Y. , Pan, H. , Guan, X. , Li, J. , Shi, J. , Dong, L. , Li, Z. , Di, H. , & Xu, J. (2016). Warmer and drier conditions alter the nitrifier and denitrifier communities and reduce N_2_O emissions in fertilized vegetable soils. Agriculture, Ecosystems & Environment, 231, 133–142.

[gcb16747-bib-0083] Zhang, D. , Wang, H. , Pan, J. , Luo, J. , Liu, J. , Gu, B. , Liu, S. , Zhai, L. , Lindsey, S. , Zhang, Y. , Lei, Q. , Wu, S. , Smith, P. , & Liu, H. (2018). Nitrogen application rates need to be reduced for half of the rice paddy fields in China. Agriculture, Ecosystems & Environment, 265, 8–14.

[gcb16747-bib-0084] Zikria, R. , & Damayanti, A. (2019). Impact of agricultural extension and risk preference on fertilizer overuse in Rice. Jurnal Agro Ekonomi, 37, 79–94. 10.21082/jae.v37n1.2019.79-94

