# Supplementary material for: CH4 and N2O emissions from smallholder agricultural systems on tropical peatlands in Southeast Asia
Source: Glob Chang Biol. 2023 May 15;29(15):4279–97. doi: 10.1111/gcb.16747 (PMC10946781; doi:10.1111/gcb.16747)
Supplement: Supplementary file 1 — Data S1 [file GCB-29-4279-s001.docx]

# **Supporting information**

**Table S1** Number of farmers using different types of soil amendments/fertilisers in the four study regions*.

| **Soil management** | **North Selangor** | **South Selangor** | **West Kalimantan** | **Central Kalimantan** | **Total** |
| --- | --- | --- | --- | --- | --- |
| **Nothing** | 2 | 8 | 0 | 10 | **20** |
| **Ash** | 7 |  | 7 | 1 | **15** |
| **Lime** | 0 | 0 | 6 | 4 | **10** |
| **Manure** | 0 | 0 | 3 | 1 | **4** |
| **Mineral soil** | 3 |  |  | 1 | **4** |
| **Lime-nutrient mixture** | 0 |  | 4 | 62 | **66** |
| **Inorganic fertiliser** | 7 | 0 | 2 |  | **9** |
| **Compost** | 5 |  | 1 | 2 | **8** |

*As part of the wider study this research is part of we interviewed farmers regarding their fertiliser inputs

**Table S2** Extended site description. Age of the oil palm plantation in 2018 is represented by the number in brackets.

| **Country** | **Region** | **Land-use class** | **Vegetation group** | **Site code** | **Coordinates** | | **Peat depth (cm)** |
| --- | --- | --- | --- | --- | --- | --- | --- |
|  |  |  |  |  | **N** | **E** |  |
| **Malaysia** | **North Selangor (NS)** | Oil palm | Oil palm | OP[16]1 | 3.42560 | 101.30673 | 40-70 |
|  |  | Oil palm | Oil palm | OP[16]2 | 3.42626 | 101.31117 | 118-141 |
|  |  | Oil palm | Oil palm | OP[16]3 | 3.42598 | 101.31861 | 117-162 |
|  |  | Cropland | Winter melon | WM1 | 3.42684 | 101.29748 | 18-20 |
|  |  | Cropland | Winter melon | WM2 | 3.42686 | 101.29815 | 10-40 |
|  |  | Cropland | Winter melon | WM3 | 3.42674 | 101.29997 | 20-40 |
|  |  | Cropland | Pineapple | PA1 | 3.42490 | 101.30568 | 20-22 |
|  |  | Cropland | Pineapple | PA2 | 3.42455 | 101.30621 | 8-47 |
|  |  | Cropland | Pineapple | PA3 | 3.42218 | 101.31077 | 40-60 |
|  |  | Forest | Forest NS | Fns1 | 3.42841 | 101.30142 | 151-176 |
|  |  | Forest | Forest NS | Fns2 | 3.42712 | 101.31118 | 200-218 |
|  |  | Forest | Forest NS | Fns3 | 3.44308 | 101.28286 | 131-142 |
| **Malaysia** | **South Selangor (SS)** | Oil palm | Oil palm | OP[0]1 | 2.74954 | 101.54572 | 20-30 |
|  |  | Oil palm | Oil palm | OP[0]2 | 2.74863 | 101.54590 | 40-50 |
|  |  | Oil palm | Oil palm | OP[0]3 | 2.74568 | 101.54552 | 98-134 |
|  |  | Cropland | Turmeric | TM1 | 2.74089 | 101.55884 | 40-50 |
|  |  | Cropland | Turmeric | TM2 | 2.74245 | 101.56011 | 20-40 |
|  |  | Cropland | Turmeric | TM3 | 2.73975 | 101.56102 | 45-73 |
|  |  | Cropland | Banana | BN1 | 2.74254 | 101.55934 | 60-77 |
|  |  | Cropland | Banana | BN2 | 2.74259 | 101.56112 | 25-50 |
|  |  | Cropland | Banana | BN3 | 2.73997 | 101.56220 | 70-86 |
|  |  | Forest | Forest SS | Fss1 | 2.68119 | 101.59746 | 150-182 |
|  |  | Forest | Forest SS | Fss2 | 2.68026 | 101.61803 | 250-300 |
|  |  | Forest | Forest SS | Fss3 | 2.70470 | 101.63114 | 200-250 |
| **Indonesia** | **West Kalimantan** | Oil palm | Oil palm | OP[4]1 | -0.3816 | 109.560 | 450-550 |
|  |  | Oil palm | Oil palm | OP[4]2 | -0.3818 | 109.563 | 478-488 |
|  |  | Oil palm | Oil palm | OP[4]3 | -0.4021 | 109.5807 | 36-40 |
|  |  | Tree plantation | Rubber | Rb1 | -0.3803 | 109.5934 | 70-75 |
|  |  | Tree plantation | Rubber | Rb2 | -0.3806 | 109.5913 | 114-150 |
|  |  | Tree plantation | Rubber | Rb3 | -0.3810 | 109.5618 | 400-450 |
|  |  | Cropland | Ginger | GG2 | -0.3801 | 109.593 | 50-60 |
|  |  | Cropland | Ginger | GG3 | -0.4011 | 109.5789 | 18-30 |
|  |  | Cropland | Ginger | GG3 | -0.4005 | 109.5809 | 118-140 |
|  |  | Forest | Forest WK | Fwk1 | -0.4019 | 109.5772 | 100-150 |
|  |  | Forest | Forest WK | Fwk2 | -0.4022 | 109.5778 | 450-500 |
|  |  | Forest | Forest WK | Fwk3 | -0.4000 | 109.5801 | 403-439 |
| **Indonesia** | **Central Kalimantan** | Oil palm | Oil palm | OP[9]1 | -2.3000 | 114.032 | 377-393 |
|  |  | Oil palm | Oil palm | OP[9]2 | -2.3556 | 114.102 | 300-312 |
|  |  | Oil palm | Oil palm | OP[9]3 | -1.9199 | 113.5786 | 231-250 |
|  |  | Tree plantation | Jelutung | J1 | -2.2893 | 114.0103 | 284-300 |
|  |  | Tree plantation | Jelutung | J2 | -2.3541 | 114.1004 | 231-250 |
|  |  | Tree plantation | Jelutung | J3 | -1.8973 | 113.5262 | 110-150 |
|  |  | Cropland | Water spinach | WS1 | -2.2892 | 114.0135 | 365-377 |
|  |  | Cropland | Water spinach | WS2 | -2.3006 | 114.0298 | 292-300 |
|  |  | Cropland | Water spinach | WS3 | -2.3085 | 114.0265 | 407-429 |
|  |  | Forest | Forest CK | Fck1 | -2.3526 | 114.0923 | 300-335 |
|  |  | Forest | Forest CK | Fck2 | -2.3521 | 114.0935 | 276-284 |
|  |  | Forest | Forest CK | Fck3 | -1.8845 | 113.4734 | 83-183 |

**Table S3** Descriptive summary statistics consisting of mean and standard error of the mean (SE) of CH_4_ and N_2_O fluxes, water table depth (WTD), total dissolved nitrogen (TDN) and soil temperature at 10 cm soil depth (T10) for each study site — Central Kalimantan (CK), West Kalimantan (WK), South Selangor (SS) and North Selangor (NS). Study sites fell into four broad land use classes: forest (F), tree plantation, including jelutung (J) and rubber (RB), oil palm (OP) of several ages (represented by the number in brackets) and cropland, including banana (BN), ginger (GG), pineapple (PA), turmeric (TM), winter melon (WM) and water spinach (WS). n is the number of samples used to calculate the mean and the SE.

| **Region** | **Land use** | **Site code** | **CH_4_ (µg m^-2^ h^-1^)*** | | | **N_2_O (µg m^-2^ h^-1^)*** | | | **WTD (cm)** | | | **TDN (mg L^-1^)** | | | **T10 (^o^C)** | | |
| --- | --- | --- | --- | --- | --- | --- | --- | --- | --- | --- | --- | --- | --- | --- | --- | --- | --- |
|  |  |  | **Mean** | **SE** | **n** | **Mean** | **SE** | **n** | **Mean** | **SE** | **n** | **Mean** | **SE** | **n** | Mean | SE | **n** |
| **CK** | **Forest** | **Fck1** | 550 | 234 | 36 | 0.2 | 6 | 22 | -20 | 4 | 36 | 1.8 | 0.1 | 11 | 26.1 | 0.1 | 36 |
| **CK** | **Forest** | **Fck2** | -5 | 10 | 38 | 6 | 5 | 22 | -31 | 2 | 38 | 1.6 | 0.1 | 11 | 26.1 | 0.1 | 38 |
| **CK** | **Forest** | **Fck3** | 3597 | 410 | 42 | 15 | 12 | 22 | -2 | 2 | 42 | 1.1 | 0.1 | 11 | 26.5 | 0.1 | 42 |
| **NS** | **Forest** | **Fns1** | 1950 | 269 | 3 | n.d | n.d | n.d | -5 | 5 | 14 | 2.7 | 0.1 | 5 | 25.8 | 0.2 | 14 |
| **NS** | **Forest** | **Fns2** | 1441 | 102 | 4 | n.d | n.d | n.d | -9 | 6 | 14 | 2.8 | 0.2 | 5 | 25.7 | 0.2 | 14 |
| **NS** | **Forest** | **Fns3** | 1896 | 1921 | 4 | n.d | n.d | n.d | -7 | 5 | 14 | 3.3 | 0.0 | 5 | 25.9 | 0.3 | 14 |
| **WK** | **Forest** | **Fwk1** | 26 | 15 | 34 | 102 | 51 | 24 | -34 | 4 | 34 | 2.1 | 0.1 | 11 | 26.8 | 0.1 | 34 |
| **WK** | **Forest** | **Fwk2** | 14 | 12 | 34 | 177 | 56 | 24 | -39 | 5 | 34 | 3.1 | 0.3 | 11 | 26.9 | 0.1 | 34 |
| **WK** | **Forest** | **Fwk3** | -11 | 10 | 34 | 148 | 46 | 24 | -43 | 3 | 34 | 2.7 | 0.3 | 11 | 27.0 | 0.1 | 34 |
| **SS** | **Forest** | **Fss1** | -50 | 48 | 6 | n.d | n.d | n.d | -18 | 4 | 16 | 2.9 | 0.2 | 5 | 26.4 | 0.0 | 16 |
| **SS** | **Forest** | **Fss2** | 253 | 77 | 6 | n.d | n.d | n.d | -17 | 6 | 16 | 3.3 | 0.2 | 5 | 25.9 | 0.1 | 16 |
| **SS** | **Forest** | **Fss3** | 24 | 8 | 6 | n.d | n.d | n.d | -26 | 4 | 16 | 3.7 | 0.3 | 5 | 26.4 | 0.1 | 16 |
| **CK** | **Tree** | **J1** | 42 | 29 | 36 | 22 | 6 | 22 | -41 | 5 | 36 | 1.8 | 0.3 | 11 | 27.9 | 0.2 | 36 |
| **CK** | **Tree** | **J2** | -19 | 18 | 36 | 9 | 2 | 22 | -60 | 5 | 36 | 2.1 | 0.2 | 11 | 26.9 | 0.1 | 36 |
| **CK** | **Tree** | **J3** | 45 | 29 | 36 | 11 | 4 | 22 | -18 | 3 | 36 | 1.1 | 0.1 | 11 | 26.6 | 0.1 | 36 |
| **WK** | **Tree** | **Rb1** | 71 | 16 | 34 | 21 | 4 | 24 | -41 | 3 | 34 | 2.8 | 0.1 | 11 | 26.6 | 0.1 | 34 |
| **WK** | **Tree** | **Rb2** | 15 | 9 | 34 | 89 | 30 | 24 | -47 | 3 | 34 | 2.6 | 0.2 | 11 | 26.9 | 0.1 | 34 |
| **WK** | **Tree** | **Rb3** | -9 | 6 | 34 | 71 | 35 | 24 | -37 | 4 | 34 | 2.4 | 0.1 | 11 | 27.4 | 0.1 | 34 |
| **SS** | **Oil palm** | **OP[0]1** | 5 | 15 | 26 | 1152 | 724 | 14 | -59 | 9 | 24 | 3.2 | 0.9 | 7 | 29.4 | 0.3 | 24 |
| **SS** | **Oil palm** | **OP[0]2** | 12 | 23 | 25 | 35 | 11 | 14 | -60 | 4 | 23 | 3.0 | 0.3 | 7 | 28.6 | 0.2 | 23 |
| **SS** | **Oil palm** | **OP[0]3** | 75 | 28 | 25 | 1253 | 539 | 14 | -38 | 4 | 23 | 7.8 | 1.4 | 7 | 28.9 | 0.3 | 23 |
| **WK** | **Oil palm** | **OP[4]1** | -0.1 | 7 | 34 | 46 | 14 | 24 | -45 | 3 | 34 | 2.1 | 0.1 | 11 | 27.9 | 0.3 | 34 |
| **WK** | **Oil palm** | **OP[4]2** | 42 | 12 | 34 | 44 | 5 | 24 | -54 | 4 | 34 | 2.8 | 0.2 | 11 | 27.8 | 0.2 | 34 |
| **WK** | **Oil palm** | **OP[4]3** | 29 | 10 | 34 | 178 | 69 | 24 | -32 | 4 | 34 | 2 | 0.2 | 11 | 27.6 | 0.2 | 34 |
| **CK** | **Oil palm** | **OP[9]1** | 10 | 14 | 36 | 8 | 3 | 22 | -75 | 6 | 36 | 1.5 | 0.2 | 11 | 27.9 | 0.1 | 36 |
| **CK** | **Oil palm** | **OP[9]2** | -3 | 12 | 36 | 15 | 4 | 22 | -46 | 4 | 36 | 1.7 | 0.1 | 11 | 28.0 | 0.2 | 36 |
| **CK** | **Oil palm** | **OP[9]3** | 24 | 8 | 36 | 11 | 2 | 22 | -22 | 3 | 36 | 1.1 | 0.1 | 11 | 27.1 | 0.1 | 36 |
| **NS** | **Oil palm** | **OP[16]1** | 21 | 10 | 26 | 210 | 103 | 14 | -40 | 1 | 24 | 3.3 | 0.8 | 7 | 26.6 | 0.1 | 24 |
| **NS** | **Oil palm** | **OP[16]2** | 61 | 21 | 26 | 11 | 10 | 14 | -26 | 1 | 24 | 2.3 | 0.4 | 7 | 26.9 | 0.1 | 24 |
| **NS** | **Oil palm** | **OP[16]3** | 17 | 13 | 26 | 43 | 26 | 14 | -41 | 3 | 24 | 3.1 | 0.5 | 7 | 27.1 | 0.1 | 24 |
| **SS** | **Cropland** | **BN1** | 6 | 7 | 26 | 545 | 334 | 14 | -35 | 5 | 24 | 8.6 | 1.9 | 7 | 28.3 | 0.3 | 24 |
| **SS** | **Cropland** | **BN2** | 118 | 62 | 26 | 100 | 51 | 14 | -52 | 5 | 24 | 4.0 | 0.7 | 7 | 28.7 | 0.3 | 24 |
| **SS** | **Cropland** | **BN3** | 364 | 163 | 26 | 73 | 22 | 14 | -32 | 4 | 22 | 3.6 | 0.3 | 7 | 28.4 | 0.1 | 22 |
| **WK** | **Cropland** | **GG1** | 174 | 105 | 34 | 33 | 6 | 24 | -21 | 3 | 34 | 2.2 | 0.1 | 11 | 28.4 | 0.1 | 34 |
| **WK** | **Cropland** | **GG2** | 65 | 26 | 34 | 251 | 45 | 24 | -29 | 2 | 34 | 2.5 | 0.1 | 11 | 27.8 | 0.2 | 34 |
| **WK** | **Cropland** | **GG3** | 28 | 10 | 34 | 177 | 39 | 24 | -29 | 2 | 34 | 2.3 | 0.2 | 11 | 28.0 | 0.1 | 34 |
| **NS** | **Cropland** | **PA1** | 44 | 27 | 24 | 43 | 12 | 14 | -50 | 9 | 24 | 4.7 | 1.2 | 7 | 28.4 | 0.2 | 24 |
| **NS** | **Cropland** | **PA2** | 21 | 12 | 26 | 89 | 16 | 14 | -55 | 10 | 24 | 37.3 | 18.0 | 7 | 28.0 | 0.2 | 24 |
| **NS** | **Cropland** | **PA3** | --27 | 14 | 26 | 264 | 81 | 14 | -41 | 4 | 24 | 3.4 | 0.3 | 7 | 27.6 | 0.1 | 24 |
| **SS** | **Cropland** | **TM1** | 58 | 41 | 24 | 1112 | 639 | 14 | -66 | 5 | 22 | 17.4 | 8.0 | 7 | 28.4 | 0.2 | 22 |
| **SS** | **Cropland** | **TM2** | 18 | 8 | 26 | 1001 | 553 | 14 | -57 | 4 | 24 | 9.2 | 1.6 | 7 | 28.3 | 0.2 | 24 |
| **SS** | **Cropland** | **TM3** | 32 | 16 | 26 | 795 | 322 | 14 | -55 | 4 | 24 | 14.4 | 7.3 | 7 | 28.6 | 0.3 | 24 |
| **NS** | **Cropland** | **WM1** | 39 | 28 | 26 | 474 | 73 | 14 | -23 | 2 | 24 | 7.0 | 1.1 | 7 | 29.4 | 0.3 | 24 |
| **NS** | **Cropland** | **WM2** | 64 | 29 | 26 | 319 | 125 | 14 | -12 | 1 | 24 | 3.0 | 0.4 | 7 | 29.5 | 0.3 | 24 |
| **NS** | **Cropland** | **WM3** | 213 | 72 | 26 | 3 | 4 | 14 | -18 | 1 | 24 | 3.2 | 0.4 | 7 | 29.3 | 0.2 | 24 |
| **CK** | **Cropland** | **WS1** | 13 | 10 | 35 | 185 | 50 | 11 | -33 | 5 | 35 | 3.5 | 0.6 | 11 | 29.5 | 0.2 | 35 |
| **CK** | **Cropland** | **WS2** | 20 | 10 | 36 | 557 | 152 | 11 | -36 | 6 | 36 | 6.2 | 1.1 | 11 | 29.5 | 0.2 | 36 |
| **CK** | **Cropland** | **WS3** | 28 | 8 | 36 | 876 | 187 | 11 | -47 | 2 | 36 | 2.2 | 0.4 | 11 | 29.4 | 0.2 | 36 |

n.d. not determined

* Average values presented in the main text in the paper are in mg m^-2^ h^-1^ and they were rounded to one significant figure.

**Table S4** Extended statistical report of effects of different the fixed terms on CH_4_ and N_2_O fluxes using Mixed Models (REML) repeated measurements including non-significant effects. Vegetation stands for vegetation group, i.e. the specific crop type, oil palm plantation type of a certain age, plantation with different tree species, or forest in one of the four study regions. Loc = location (near the crop/stem vs. far from the crop/stem), wtd= water table depth, TDN = total dissolved nitrogen, T10 = soil temperature at 10 cm soil depth. Variables followed by BxC means that a box cox transformation of the data was performed prior to the statistical analysis. Significant results are highlighted in bold

| **Fixed term** | **CH4 fluxes** | | | **N2O fluxes** | | | | |
| --- | --- | --- | --- | --- | --- | --- | --- | --- |
|  | **Wald statistic** | **d.f.** | **Wald / d.f.** | **chi pr** | **Wald statistic** | **d.f.** | **Wald / d.f.** | **chi pr** |
| **vegetation** | **35.01** | **13** | **2.69** | **<0.001** | **61.27** | **13** | **4.71** | **<0.001** |
| loc | 0.67 | 1 | 0.67 | 0.414 | **9** | **1** | **9** | **0.003** |
| *T10_BxC* | *3.35* | *1* | *3.35* | *0.067* | 2.42 | 1 | 2.42 | 0.119 |
| **wtd_BxC** | **7.65** | **1** | **7.65** | **0.006** | **12.89** | **1** | **12.89** | **<0.001** |
| TDN_BxC | 2.39 | 1 | 2.39 | 0.122 | **17.67** | **1** | **17.67** | **<0.001** |
| *vegetation.loc* | *20.37* | *13* | *1.57* | *0.086* | **41.64** | **13** | **3.2** | **<0.001** |
| **vegetation.T10_BxC** | **23.11** | **13** | **1.78** | **0.040** | **52.49** | **13** | **4.04** | **<0.001** |
| loc.T10_BxC | 0.66 | 1 | 0.66 | 0.417 | 1.75 | 1 | 1.75 | 0.186 |
| **vegetation.wtd_BxC** | **28.44** | **13** | **2.19** | **0.008** | **49.45** | **13** | **3.8** | **<0.001** |
| loc.wtd_BxC | 0.57 | 1 | 0.57 | 0.452 | 0.17 | 1 | 0.17 | 0.678 |
| T10_BxC.wtd_BxC | 1.31 | 1 | 1.31 | 0.253 | *2.85* | *1* | *2.85* | *0.092* |
| vegetation.TDN_BxC | 11.03 | 13 | 0.85 | 0.608 | **33.57** | **13** | **2.58** | **0.001** |
| loc.TDN_BxC | 0.7 | 1 | 0.7 | 0.401 | 1.09 | 1 | 1.09 | 0.297 |
| T10_BxC.TDN_BxC | 0.2 | 1 | 0.2 | 0.655 | *3.53* | *1* | *3.53* | *0.060* |
| wtd_BxC.TDN_BxC | 0.45 | 1 | 0.45 | 0.502 | 0.67 | 1 | 0.67 | 0.413 |
| vegetation.loc.T10_BxC | 11.85 | 13 | 0.91 | 0.540 | 15.56 | 13 | 1.2 | 0.274 |
| **vegetation.loc.wtd_BxC** | **23.03** | **13** | **1.77** | **0.041** | **25.68** | **13** | **1.98** | **0.019** |
| *vegetation.T10_BxC.wtd_BxC* | *22.16* | *13* | *1.7* | *0.053* | 14.76 | 13 | 1.14 | 0.322 |
| *loc.T10_BxC.wtd_BxC* | *2.62* | *1* | *2.62* | *0.106* | 1.72 | 1 | 1.72 | 0.189 |
| vegetation.loc.TDN_BxC | 5.8 | 13 | 0.45 | 0.953 | 18.02 | 13 | 1.39 | 0.157 |
| **vegetation.T10_BxC.TDN_BxC** | **30.05** | **13** | **2.31** | **0.005** | **34.71** | **13** | **2.67** | **<0.001** |
| loc.T10_BxC.TDN_BxC | 0.02 | 1 | 0.02 | 0.901 | **7.83** | **1** | **7.83** | **0.005** |
| vegetation.wtd_BxC.TDN_BxC | 13.1 | 13 | 1.01 | 0.440 | 19.16 | 13 | 1.47 | 0.118 |
| loc.wtd_BxC.TDN_BxC | 0.46 | 1 | 0.46 | 0.497 | 0.07 | 1 | 0.07 | 0.798 |
| T10_BxC.wtd_BxC.TDN_BxC | 1.69 | 1 | 1.69 | 0.194 | 0.47 | 1 | 0.47 | 0.495 |
| vegetation.loc.T10_BxC.wtd_BxC | 13.44 | 13 | 1.03 | 0.414 | 11.28 | 13 | 0.87 | 0.588 |
| vegetation.loc.T10_BxC.TDN_BxC | 16.46 | 13 | 1.27 | 0.225 | 12.84 | 13 | 0.99 | 0.460 |
| vegetation.loc.wtd_BxC.TDN_BxC | 9.17 | 13 | 0.71 | 0.760 | 6.73 | 13 | 0.52 | 0.916 |
| vegetation.T10_BxC.wtd_BxC.TDN_BxC | 7.1 | 13 | 0.55 | 0.897 | **28.73** | **13** | **2.21** | **0.007** |
| loc.T10_BxC.wtd_BxC.TDN_BxC | 1.79 | 1 | 1.79 | 0.182 | 2.01 | 1 | 2.01 | 0.156 |
| vegetation.loc.T10_BxC.wtd_BxC.TDN_BxC | 9.09 | 13 | 0.7 | 0.766 | 19.34 | 13 | 1.49 | 0.113 |


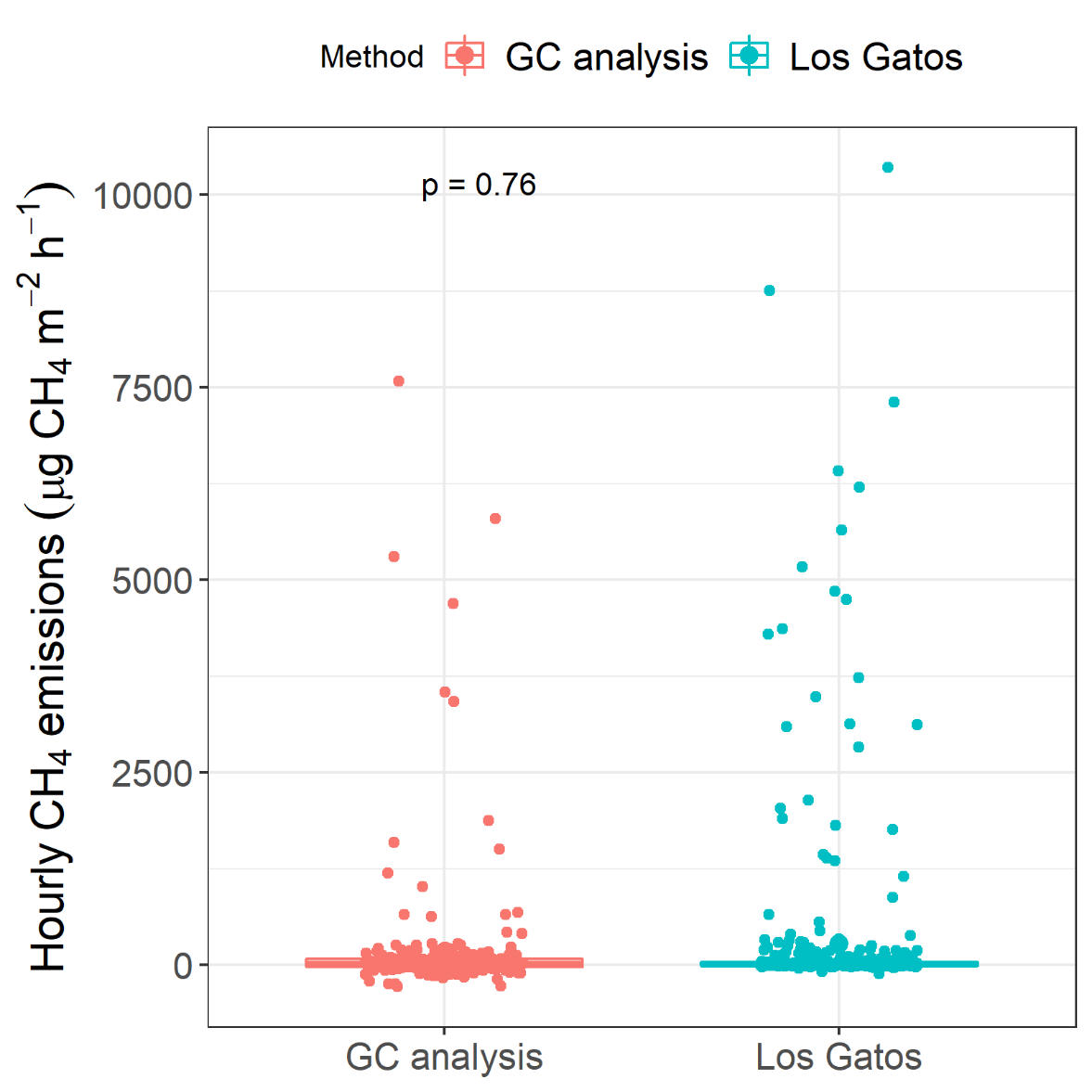


**Figure S1** Comparison of CH_4_ emissions determined by GC analysis and by Los Gatos analyser.


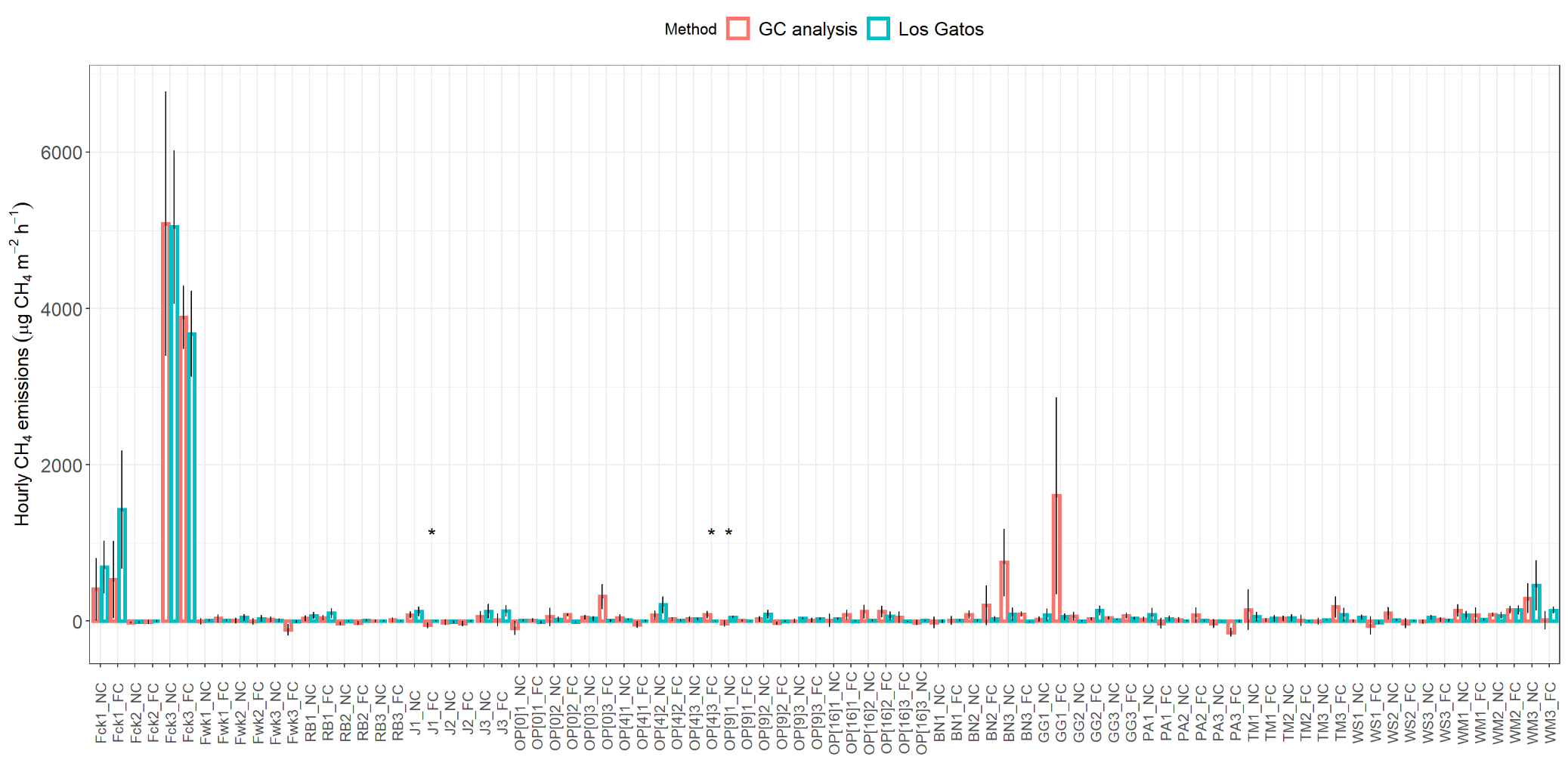


**Figure S2** Comparison of CH_4_ emissions determined by GC analysis and by Los Gatos analyser measured either near the crop/stem (NC) or far from the crop stem (FC). Sites include Forest Central Kalimantan (Fck), Forest West Kalimantan (Fwk), Rubber (RB), Jelutung (J), Oil palm (OP) of different ages (age in brackets), Banana (BN), Ginger (GG), Pineapple (PA), Turmeric (TM), Water spinach (WS) and Winter melon (WM). Statistically significant differences between the two methods at the plot level are highlighted by an asterisk (*)

**
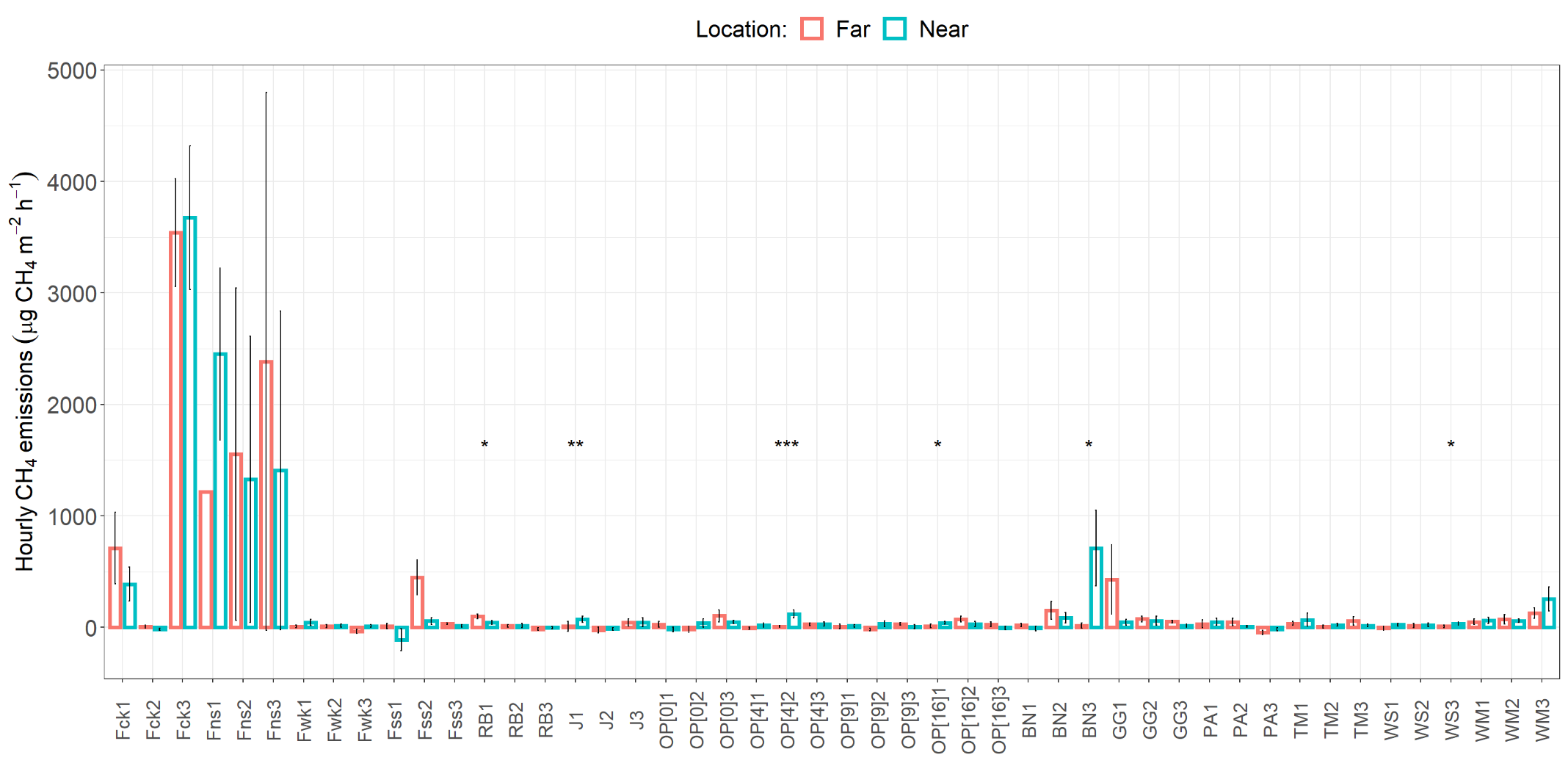
**

**Figure S3** Pair wise comparison of CH_4_ emissions measured at the near the crop/stem (Near)and far from the crop / stem (Far) locations within each study site. Sites include Forest Central Kalimantan (Fck), Forest North Selangor (Fns), Forest West Kalimantan (Fwk), Forest South Selangor (Fss), Rubber (RB), Jelutung (J), Oil palm (OP) of different ages (age in brackets), Banana (BN), Ginger (GG), Pineapple (PA), Turmeric (TM), Water spinach (WS) and Winter melon (WM). Statistically significant differences between the two locations at the site level are highlighted by an arterisk (*)


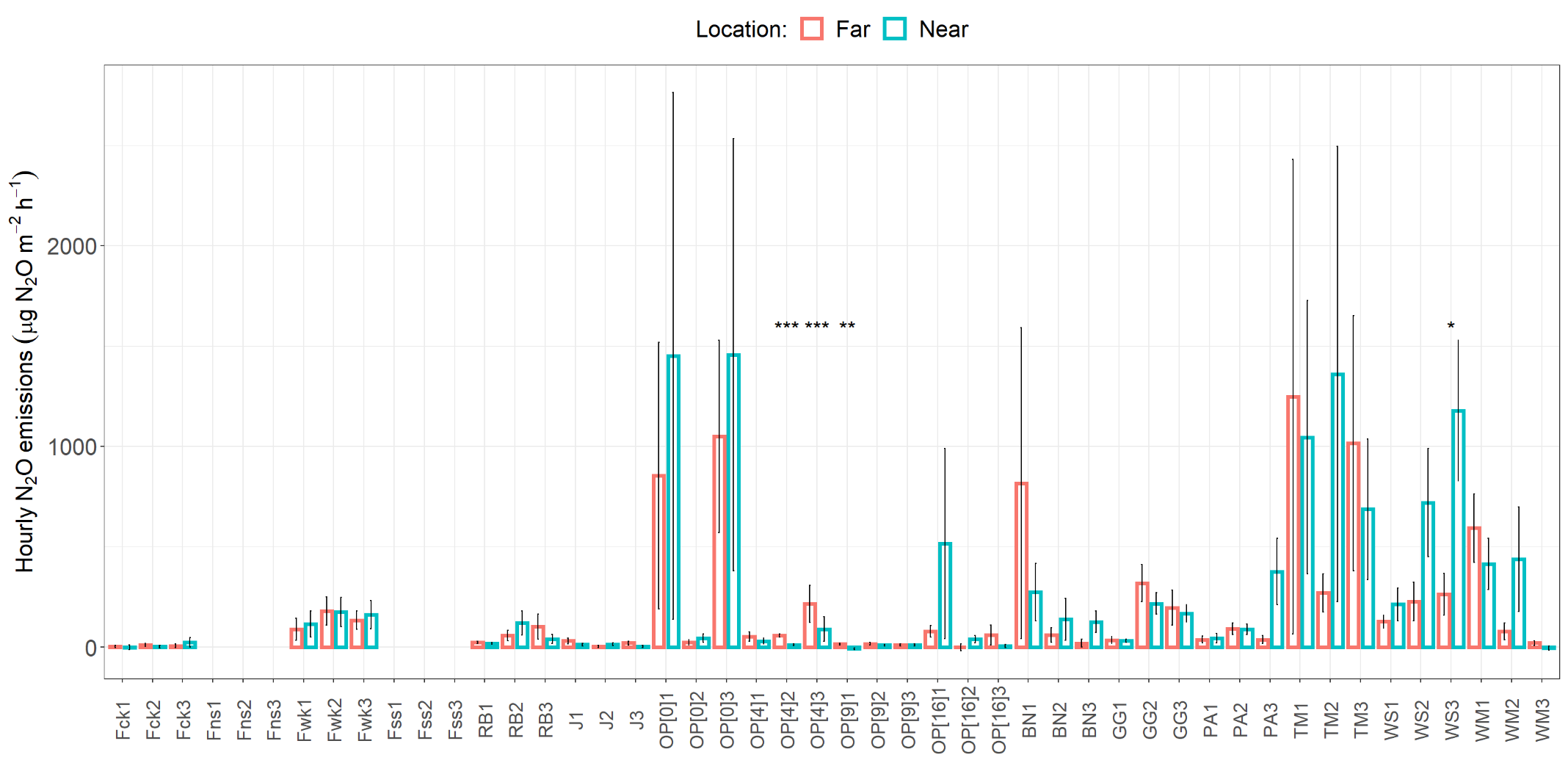


**Figure S4** Pair wise comparison of N_2_O emissions measured at the near the crop /stem (Near) and far from the crop / stem (Far) locations within each study site. Sites include Forest Central Kalimantan (Fck), Forest West Kalimantan (Fwk), Rubber (RB), Jelutung (J), Oil palm (OP) of different ages (age in brackets), Banana (BN), Ginger (GG), Pineapple (PA), Turmeric (TM), Water spinach (WS) and Winter melon (WM). No available data for the Forest North Selangor (Fns) and South Selangor (Fss) sites. Statistically significant differences between the two locations at the site level are highlighted by an arterisk (*)


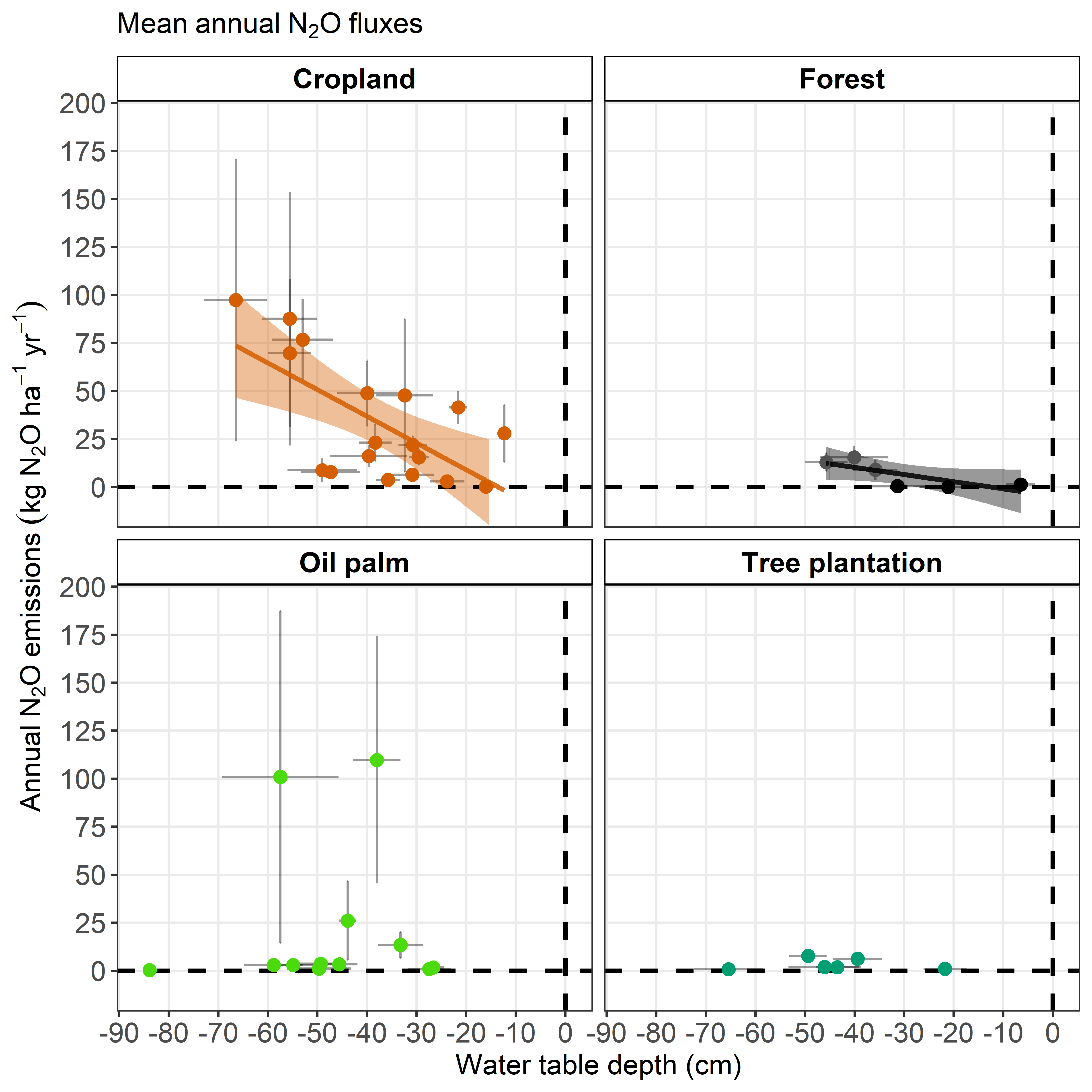


**Figure S5** Relationship between annual water table depth and N_2_O emissions for different land-use classes.
